# Annexin A13 Protects Against Acute Kidney Injury by Inactivating TGF‐β/Smad3 Signaling

**DOI:** 10.1002/advs.202504356

**Published:** 2026-01-04

**Authors:** Jiaxiao Li, Chen Wu, Yuqi Zhu, Zicheng Liu, Wenjuan Yu, Andrew Lukwaro, Yu Zhong, Guoqiang Xie, Lili Zhou, Xiaoru Huang, Hui‐yao Lan, Junzhe Chen, Ying Tang

**Affiliations:** ^1^ Department of Nephrology The Third Affiliated Hospital of Southern Medical University Guangzhou China; ^2^ Department of Medicine &Therapeutics the Chinese University of Hong Kong Hong Kong China; ^3^ Guangdong‐Hong Kong Joint Laboratory for Immunological and Genetic Kidney Disease Guangdong Academy of Medical Science Guangdong Provincial People's Hospital Guangzhou China; ^4^ State Key Laboratory of Organ Failure Research National Clinical Research Center of Kidney Disease Division of Nephrology Nanfang Hospital Southern Medical University Guangzhou China

**Keywords:** AKI, ANXA13, TGF‐β receptor type 1, TGF‐β/Smad3

## Abstract

Acute kidney injury (AKI) is a common cause of chronic kidney disease, but the underlying pathogenesis remains unclear, and treatment options are limited. Here we report that Annexin A13 (ANXA13), the founder member of Annexins, is renoprotective in AKI. Clinically, ANXA13 is lost in the kidneys of patients with AKI and in mice with ischemic‐reperfusion injury (IRI)‐ or cisplatin‐induced AKI. This was associated with reduced serum ANXA13 and elevated urinary ANXA13 levels in the patients. Functionally, ANXA13 overexpression protected against IRI‐ and cisplatin‐induced AKI, whereas ANXA13 silencing promoted AKI. This was further confirmed in renal tubule epithelial cell‐specific *Anxa13* knockout mice, in which deletion of tubular Anxa13 significantly exacerbated IRI‐ and cisplatin‐induced AKI. Mechanistically, ANXA13 directly binds to the TGF‐β receptor type 1 intracellular domain and inhibits its phosphorylation. This inactivates Smad3 signaling and blocks Smad3‐mediated tubular cell death via p21‐dependent G1 cell cycle arrest. Furthermore, our findings revealed that ANXA13 was negatively regulated by TGF‐β/Smad3 signaling, as Smad3 could bind to the 3ʹUTR of ANXA13 and inhibit its transcription, which was confirmed in *Smad3* Knockout mice. In conclusion, ANXA13 is renoprotective in AKI and may be a novel therapeutic agent for AKI by targeting TGF‐β/Smad3 signaling.

## Graphical abstract

ANXA13 is negatively regulated by Smad3 and exerts its protective role in AKI by inactivating TGF‐β/Smad3 signaling and Smad3‐p21 cell cycle arrest pathway through binding to TβRI, inhibiting the interaction between TβRI and TβRII, thereby suppressing TβRI phosporylation.

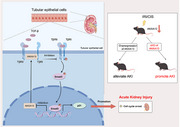

## 1 Introduction

Acute kidney injury (AKI) is a prevalent clinical syndrome characterized by sudden loss of renal function and acute tubular necrosis. It affects 10–15% of hospitalized patients and >50% of those admitted to intensive care units [1, 2]. Incomplete recovery from AKI significantly contributes to the progression of chronic kidney disease (CKD) [3]. However, the underlying mechanisms of AKI remain poorly understood, and effective therapies are lacking. Thus, a better understanding of the molecular mechanisms underlying AKI is urgently needed and is the first step toward the development of effective therapies for AKI.

The vertebrate annexin superfamily, Annexins (ANXAs), including ANXA1 to ANXA11 and ANXA13, consists of evolutionarily conserved proteins that feature a uniform core domain and a unique N‐terminal domain. Studies have demonstrated that ANXAs participate in the regulation of cellular functions including membrane transport and intracellular signaling [4, 5, 6]. Although most ANXAs are widely expressed in all tissues, the expression of ANXA13 is restricted to intestinal and renal cells [7, 8]. The renoprotective roles of ANXA1 and ANXA2 have been documented in AKI and renal fibrosis [9, 10]. However, the role of ANXA13, the founder member and prototype of the conserved evolutionary properties of the ANXAs superfamily [11, 12], in AKI remains unexplored, despite its reported involvement in cancer cell invasion and migration in vitro [13, 14].

TGF‐β/Smad3 signaling plays a pathogenic role in AKI by suppressing cell proliferation and triggering tubular epithelial cell (TEC) death, and increasing inflammation and oxidative stress [15, 16, 17]. Smad3 interacts with the promoter of the cyclin‐dependent kinase inhibitor p21/p27 to cause TEC death in AKI kidneys via G1 cell cycle arrest, which can be blocked by genetic deletion or pharmacological inhibition of *Smad3*[18]. However, the relationship between ANXA13 and TGF‐β signaling in kidney diseases remains unclear.

In this study, we first examined the expression of ANXA13 in the kidneys of patients and mice with AKI. Subsequently, we determined the functional role of ANXA13 in AKI by overexpressing ANXA13 or conditionally knocking out (cKO) Anxa13 in renal tubules. Finally, the protective mechanisms of ANXA13 related to TGF‐β/*Smad3* signaling in AKI were investigated in vivo and in vitro.

## 2 Results

### 2.1 Anxa13 is Downregulated in the Kidney in Patients and Mice With AKI

**FIGURE 1:**
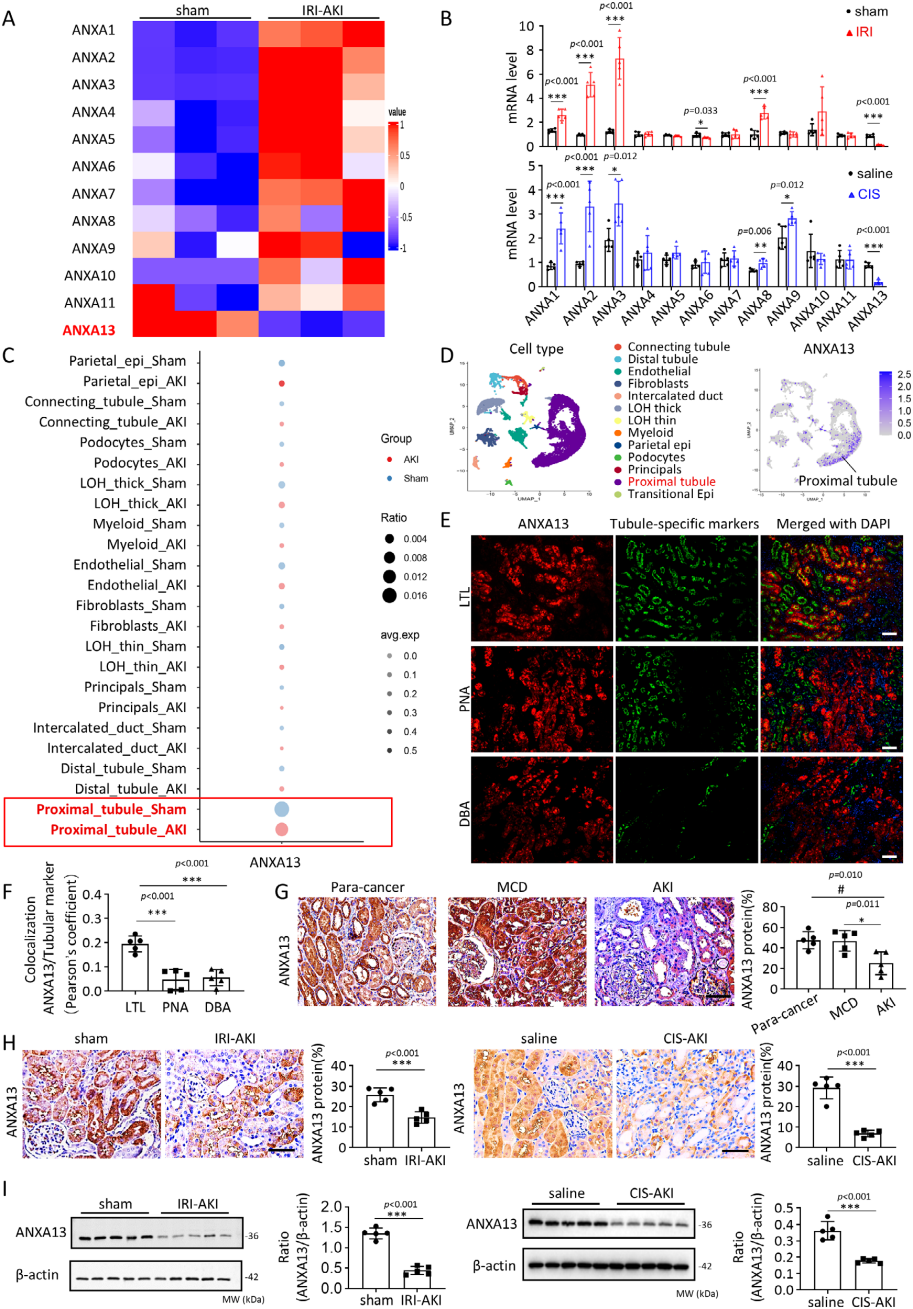
ANXA13 is downregulated in the kidneys of mice and patients with AKI. (A) Heatmap of ANXAs gene expression in sham and IRI‐induced AKI mice using RNA‐seq. (B) Relative renal mRNA levels of ANXAs in IRI‐ and cisplatin‐induced AKI mice. (C) Single‐cell RNA sequencing (scRNA‐seq) reveals ANXA13 is downregulated in proximal tubular cells in AKI. (D) Cell annotation of kidney cell subsets, the distribution, and relative expression of ANXA13 in different kidney cell subsets using scRNA‐seq showed that ANXA13 is largely expressed by proximal tubular cells in the mouse kidney. (E) Two‐color immunofluorescence shows that most ANXA13 (red) is co‐localized with LTL (green, a marker for proximal renal tubules), with few co‐localized with PNA (green, a marker for distal renal tubules) and DBA (green, a marker for collecting ducts). (F) Quantification of ANXA13 and renal tubular marker co‐localization using Pearson's coefficient. (G) Immunohistochemistry shows that ANXA13 is largely expressed in the kidneys of para‐cancer and patients with minimal change disease (MCD), but is mainly reduced in patients with AKI. (H) Immunohistochemistry shows that ANXA13 is expressed largely by proximal tubular cells in normal sham or saline mice, which is lost in IRI‐AKI and Cisplatin‐induced AKI mice. (I) Western blot analysis of ANXA13 in IRI‐ and Cisplatin‐induced AKI mice. Data are reported as the mean ± SD from group of 5 mice or patients. Statistical analysis was performed using an unpaired t‐test for B, H, and I, and one‐way ANOVA with Tukey's test for F and G. ^*^ *p* <0.05, ^***^ *p* <0.001 versus sham or Saline or MCD group or LTL; ^##^ *p* <0.01 versus para‐cancer group. Scale bars = 50 µm.

We first detected the gene expression of the ANXAs family in the kidneys of sham and ischemic‐reperfusion injury (IRI)‐induced AKI mice using RNA sequencing (RNA‐seq). Our results showed that only Anxa13 expression was significantly downregulated in the IRI‐AKI mice (Figure 1A). Real‐time PCR confirmed that among the ANXAs family members, only Anxa13 mRNA was downregulated in the kidneys of IRI‐ and cisplatin‐induced AKI mice (Figure 1B). We further characterized the expression pattern of Anxa13 in the mouse kidneys using single‐cell RNA sequencing (scRNA‐seq) analysis and observed that Anxa13 was largely expressed by proximal tubular cells in the normal kidney, but this expression was lost during AKI (Figure 1C,D, Figure S1). Two‐color immunofluorescence also revealed that ANXA13 was primarily expressed in proximal tubular cells, as demonstrated by co‐localization with the specific marker LTL (Figure 1E,F; Figure S2). In contrast, lower amounts of ANXA13 co‐localized with PNA and DBA, which are markers of the distal tubules and collecting ducts (Figure 1E,F). Similar results were reported in the human kidney using scRNA‐seq in which ANXA13, but not the other ANXAs family members, was mainly expressed by proximal tubular cells (Figure S3, From The Human Protein Atlas [19]). However, in patients with AKI, the expression of renal ANXA13 was largely decreased (Figure 1G). Further analysis using western blotting and immunohistochemistry detected decreased ANXA13 expression in IRI‐ and cisplatin‐induced AKI kidneys, presumably in the proximal tubular cells (Figure 1H,I).

### 2.2 Serum and Urine ANXA13 Levels Correlate With the Severity of Renal Impairment in Aki Patients and Mice

**FIGURE 2:**
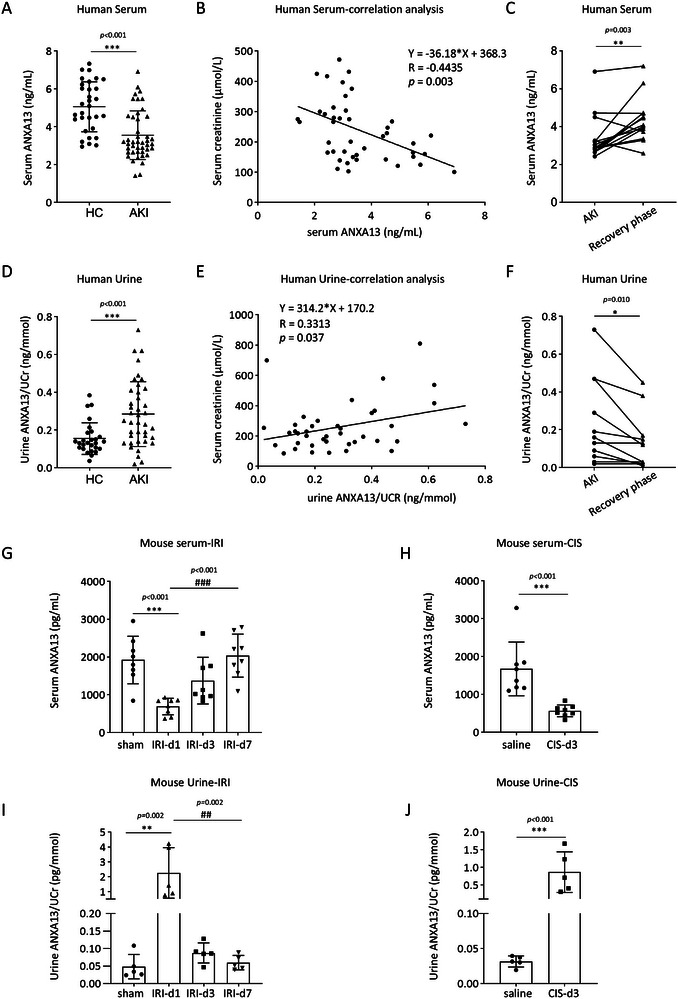
Correlation between serum/urinary ANXA13 and serum creatinine levels in patients and mice with AKI. (A) Changes in ANXA13 serum levels in patients with AKI and healthy controls (Healthy controls: n = 31; patients with AKI: n = 44). (B) Correlation between serum ANXA13 and serum creatinine levels in patients with AKI (n = 44). (C) Changes in serum ANXA13 levels in patients with AKI during the recovery phase (n = 15). (D) ANXA13 urine levels in patients with AKI and healthy controls (Healthy controls: n = 27; patients with AKI: n = 40). (E) Correlation between urinary ANXA13 and serum creatinine levels in patients with AKI (n = 40). (F) Urinary ANXA13 levels in patients with AKI during the recovery phase (n = 11). (G) Serum ANXA13 levels in IRI‐AKI mice on days 0, 1, 3, and 7 (n =8). (H) Serum ANXA13 levels in Cisplatin‐induced AKI mice on days 0 and 3 (n = 8). (I) Urinary ANXA13 levels in IRI‐AKI mice on days 0, 1, 3, and 7 (n = 5). (J) Urinary ANXA13 levels in IRI‐AKI mice on days 0 and 3 (n = 5). Data are reported as the mean ± SD from groups. Statistical analysis was performed using an unpaired t‐test for A, D, H and J, paired t‐test for C and F, simple linear regression analysis for B and C, one‐way ANOVA with Tukey's test for G and one‐way ANOVA with Dunnett's test for H. ^*^ *p* <0.05, ^**^ *p* <0.01, ^***^ *p* <0.001 versus HC or sham or Saline group, ^##^ *p*<0.01, ^###^ *p*<0.001 versus IRI d1.

Next, we measured the serum and urine ANXA13 levels in AKI patients and mice. Compared to healthy controls, serum ANXA13 levels were significantly decreased in patients with AKI and were negatively correlated with the serum creatinine (SCr) levels (Figure 2A,B). In patients in the recovery phase of AKI, serum ANXA13 levels were significantly increased (Figure 2C). In contrast, urinary ANXA13 levels were significantly elevated in patients with AKI compared to those in healthy controls, showing a positive correlation with increased SCr levels. Elevated urinary ANXA13 levels decreased during the AKI recovery phase (Figure 2D–F). These observations were further confirmed in a mouse model of IRI‐AKI, in which decreased serum ANXA13 levels were associated with increased urinary ANXA13 levels on day 1, which returned to normal within seven days (Figure 2G,I). Similarly, in mice with cisplatin‐induced AKI, decreased serum ANXA13 levels were accompanied by a concomitant increase in urinary ANXA13 levels (Figure 2H,J).

### 2.3 Overexpression of ANXA13 Protects Against but Knockdown of ANXA13 promotes IRI‐Induced AKI in Mice

**FIGURE 3:**
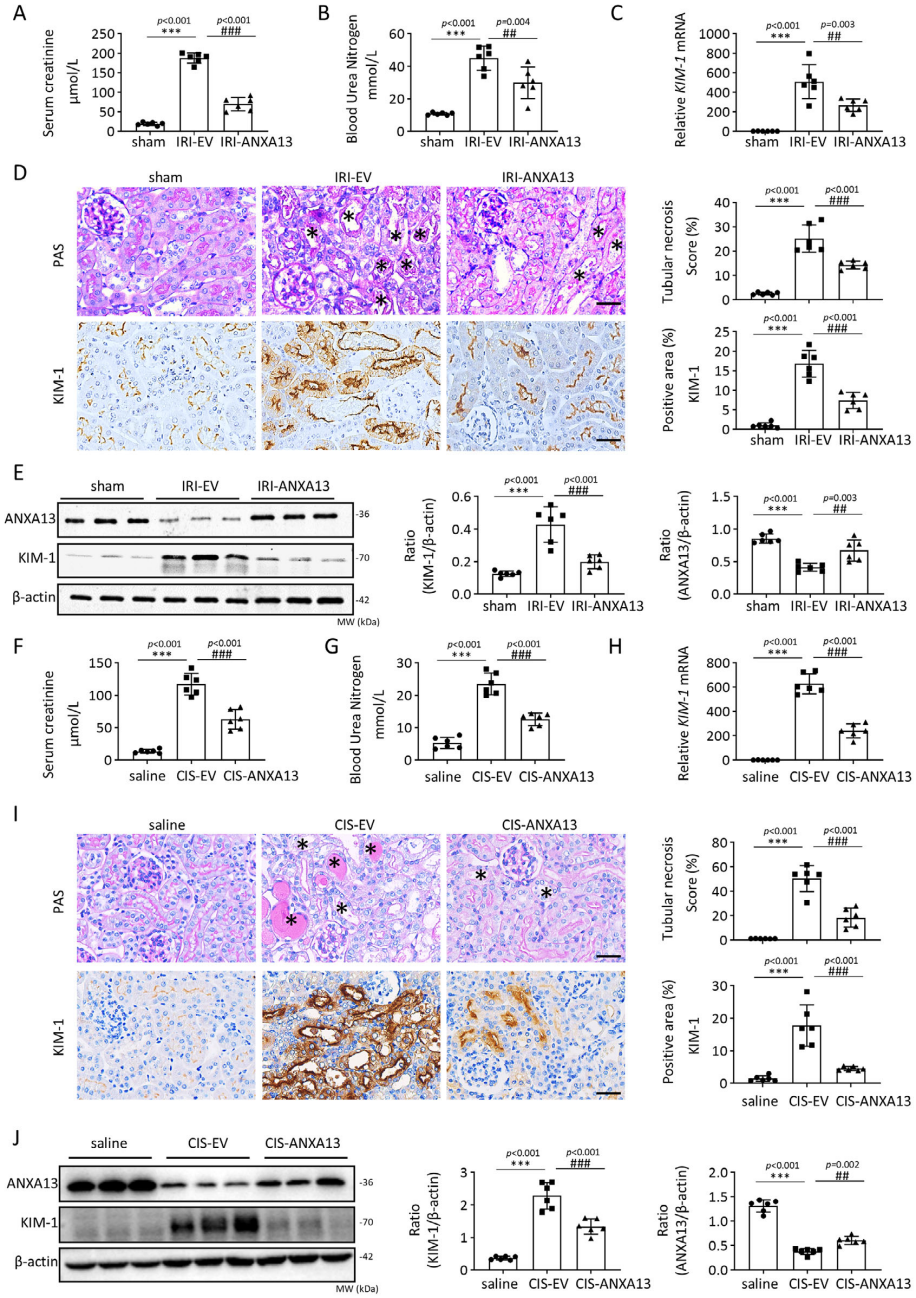
ANXA13 overexpression protects against IRI‐ and cisplatin‐induced AKI in mice. (A,B) Serum creatinine (SCr) and blood urea nitrogen (BUN) levels in IRI‐induced AKI mice. (C) Relative mRNA levels of kidney injury molecule 1 (KIM‐1) in IRI‐induced AKI mice. (D) Immunohistochemical staining of KIM‐1 and PAS staining for the detection of tubular necrosis in IRI‐induced AKI mice. (E) Western blot analyses of ANXA13 and KIM‐1 in IRI‐induced AKI mice. (F,G) SCr and BUN levels in cisplatin‐induced AKI mice. (H) Relative mRNA levels of KIM‐1 in cisplatin‐induced AKI mice. (I) Immunohistochemical staining of KIM‐1 and PAS staining for the detection of tubular necrosis in cisplatin‐induced AKI mice. (J) Western blot analysis of ANXA13 and KIM‐1 in cisplatin‐induced AKI mice. Data are reported as the mean ± SD of 6 mice per group. Statistical analysis was performed using one‐way ANOVA with Tukey's test for E (ANXA13), G, J (ANXA13), and one‐way ANOVA with Dunnett's test for A–D, E (KIM‐1), F. H, I, J (KIM‐1). ^***^ *p* <0.001 versus sham or Saline, ^##^ *p* <0.01, ^###^ *p* <0.001 versus IRI‐EV or CIS‐EV, scale bars = 50 µm, “*” indicated injured tubules.

To investigate the role of ANXA13 in AKI, we overexpressed ANXA13 (Figure S5) by transferring an ANXA13 protein‐expressing plasmid into both kidneys a day before IRI‐induced AKI using the ultrasound‐microbubble technique, as previously described [20]. As shown in Figure 3A–E, overexpression of ANXA13 significantly inhibited IRI‐induced AKI, as evidenced by reduced SCr and blood urea nitrogen (BUN) levels, suppression of tubular necrosis, and downregulation of kidney injury molecule‐1 (KIM‐1), a marker of tubular cell injury. These results revealed a protective role of ANXA13 in AKI. This novel finding was confirmed by knocking down renal ANXA13 using an ultrasound‐microbubble‐mediated ANXA13 shRNA plasmid transfer technique. As shown in Figure S6, silencing of renal ANXA13 largely exacerbated IRI‐induced AKI, leading to increased SCr and BUN levels and worsening renal tubular necrosis. Silencing renal ANXA13 increased the expression of KIM‐1 at the mRNA and protein levels in the AKI kidneys.

### 2.4 Overexpression of ANXA13 Protects Against Cisplatin‐Induced AKI in Mice

To verify the protective role of ANXA13 in AKI, we overexpressed ANXA13 using the same ultrasound‐microbubble‐mediated Anxa13 gene transfer approach in a cisplatin‐induced AKI model. Anxa13 overexpression significantly alleviated cisplatin‐induced kidney dysfunction and tubular injury, as evidenced by reduced SCr and BUN levels, and marked inhibition of tubular necrosis (Figure 3F–I). Moreover, western blotting, qPCR and immunohistochemistry demonstrated that Anxa13 overexpression significantly attenuated KIM‐1 (Figure 3I,J). Collectively, these findings suggested that ANXA13 plays a protective role in IRI‐ and cisplatin‐induced AKI.

### 2.5 Tubular Epithelial Cell‐Specific Deletion of Anxa13 Promotes IRI‐ and Cisplatin‐Induced AKI in Mice

**FIGURE 4:**
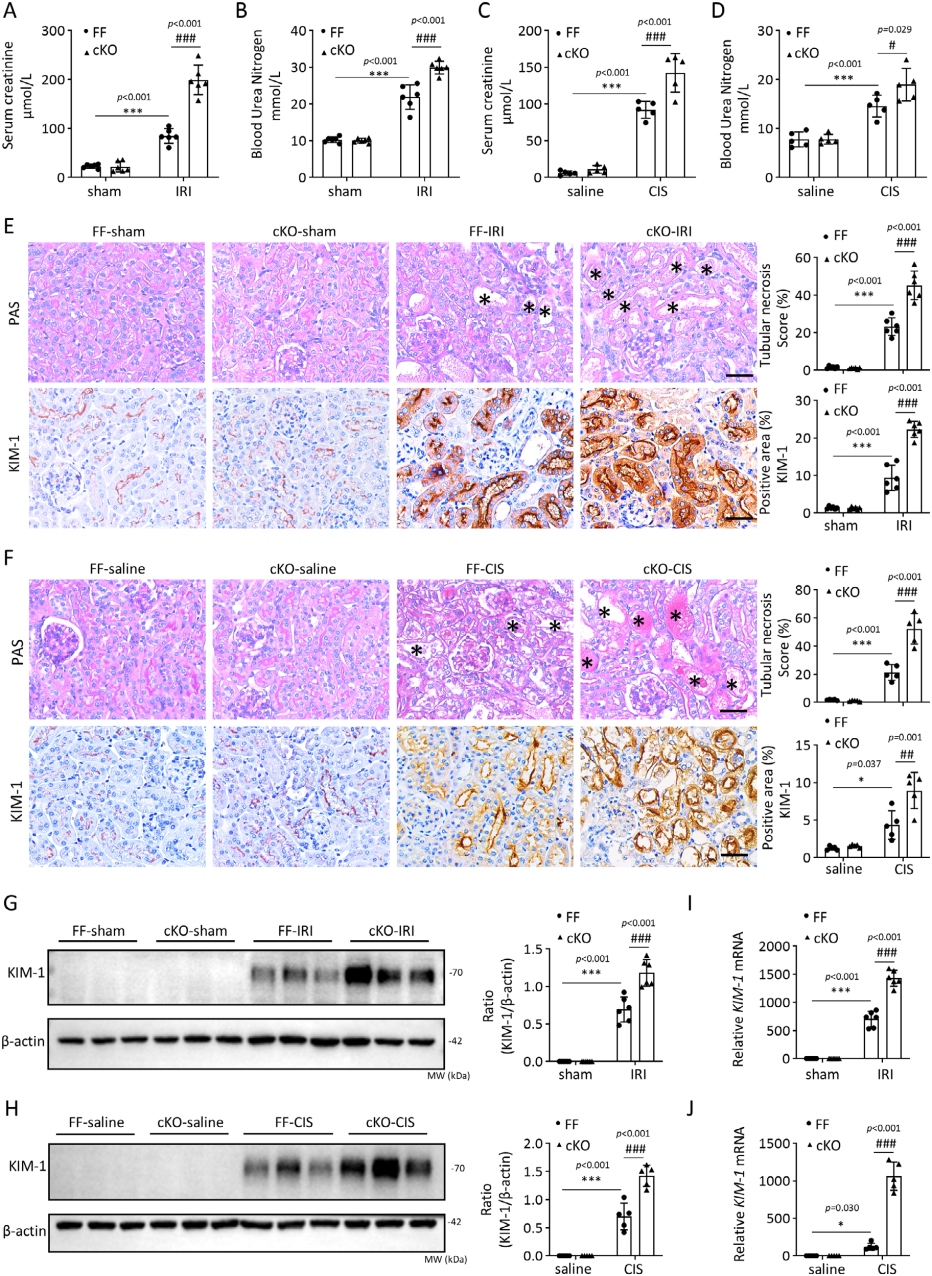
Deletion of *Anxa13* from renal tubular epithelial cells promotes IRI‐ and cisplatin‐induced AKI in mice. (A–D) SCr and BUN levels in *Anxa13* Flox/Flox (FF) and *Anxa13* Flox/Flox/Ggt1‐Cre (cKO) mice following IRI or cisplatin treatment. (E,F) Immunohistochemistry forANXA13 and KIM‐1, and PAS staining for the detection of tubular necrosis in IRI‐ or cisplatin‐induced AKI in *Anxa13* FF and cKO mice. (G,H) Western blot analysis of KIM‐1 in IRI‐ or cisplatin‐induced AKI in *Anxa13* FF and cKO mice. (I,J) Relative mRNA levels of KIM‐1 in IRI‐ or cisplatin‐induced AKI in *Anxa13* FF and cKO mice. Data are reported as mean ± SD from groups of 5–6 mice. Statistical analysis was performed using two‐way ANOVA with Tukey's test for panels A–J. ^*^ *p* <0.05, ^***^ *p* <0.001 versus FF sham or saline, ^#^ *p* <0.05, ^##^ *p* <0.01, ^###^ *p* <0.001 versus FF IRI or FF CIS. Scale bars = 50 µm; “*” indicates injured tubules.

As ANXA13 is primarily expressed by renal tubular epithelial cells, we constructed tubular epithelial cell‐specific Anxa13‐KO mice (cKO) (Figure S7) to further clarify the functional role of ANXA13 in IRI and cisplatin‐induced AKI mouse models. As shown in Figure 4, tubular‐specific deletion of Anxa13 resulted in severe AKI after IRI or cisplatin induction, as determined by a significant increase in SCr and BUN levels and the development of severe tubular damage, including necrosis and KIM‐1 expression. These results demonstrated the protective role for ANXA13 in AKI.

### 2.6 ANXA13 Protects Against AKI by Inhibiting p21‐Mediated G1 Cell Cycle Arrest via a Smad3‐Dependent Mechanism

**FIGURE 5:**
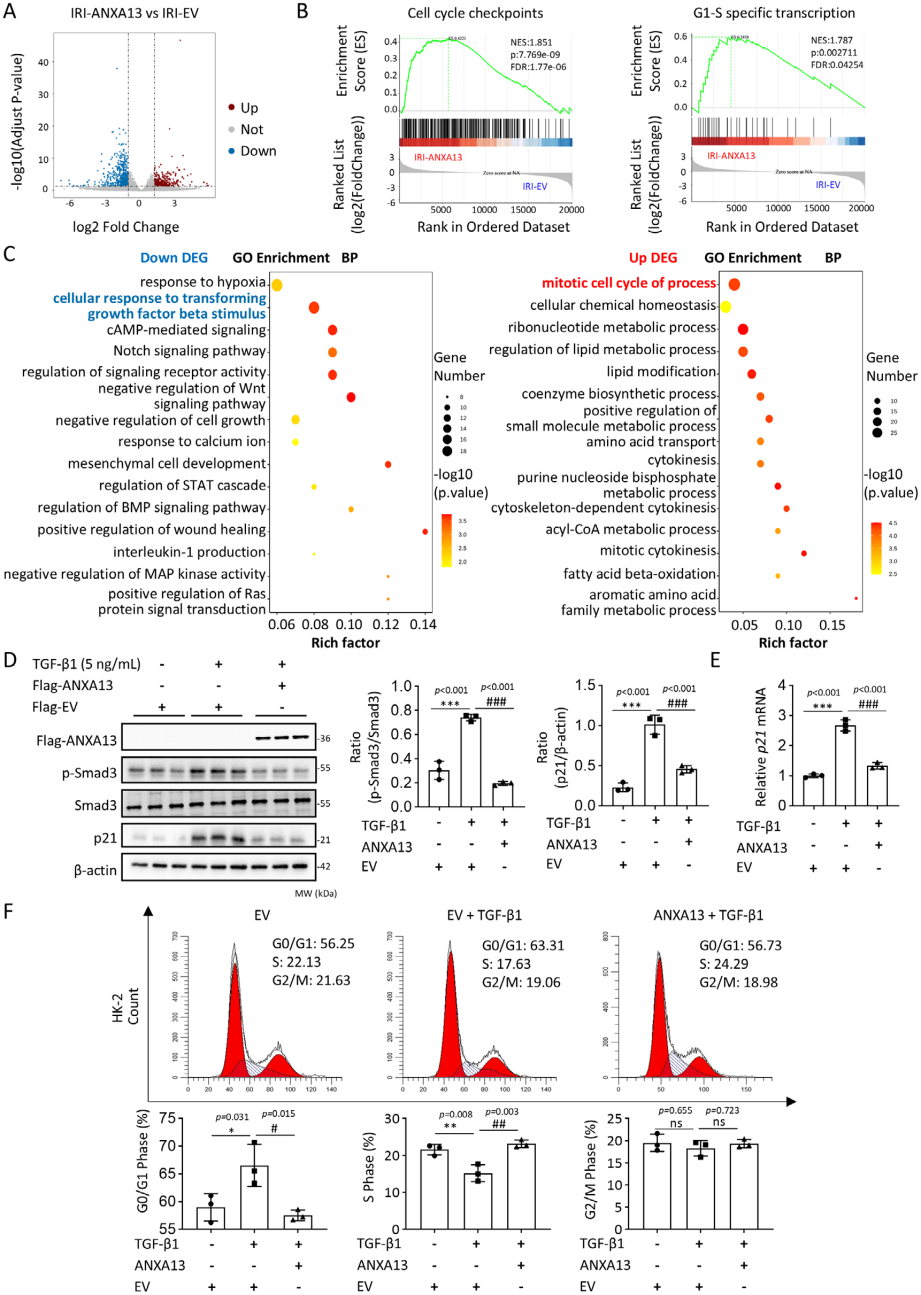
The protective effect of ANXA13 is associated with the TGF‐β/Smad3 signaling pathway. (A) Volcano plot of differentially expressed genes (DEGs) in IRI‐induced AKI mice, dots represent individual genes, with blue dots indicating downregulated DEGs and red dots indicating upregulated DEGs. (B) GSEA analyses of the cell cycle checkpoint and G1‐S specific transcription. (C) Bubble graph for Gene Ontology (GO) biological process (BP) enrichment analysis of upregulated and downregulated DEGs. (D) Western blot analysis of p‐Smad3 and p21 in HK‐2 cells overexpressing ANXA13 under TGF‐β1 (5 ng/mL) conditions. (E) Relative mRNA levels of p21 in ANXA13‐overexpressing HK‐2 cells in response to TGF‐β1 (5 ng/mL) stimulation. (F) Cell cycle analysis of HK‐2 cells treated with TGF‐β1 (5 ng/mL) using flow cytometry. Data are reported as the mean ± SD from 3 independent experiments in vitro. Statistical analysis was performed using one‐way ANOVA with Tukey's test for D–F. ^*^ *p* <0.05, ^**^ *p* <0.01, ^***^ *p* <0.001 versus Flag‐EV, ^#^ *p* <0.05, ^##^ *p* <0.01, ^###^ *p* <0.001 versus Flag‐EV with TGF‐β1.

**FIGURE 6:**
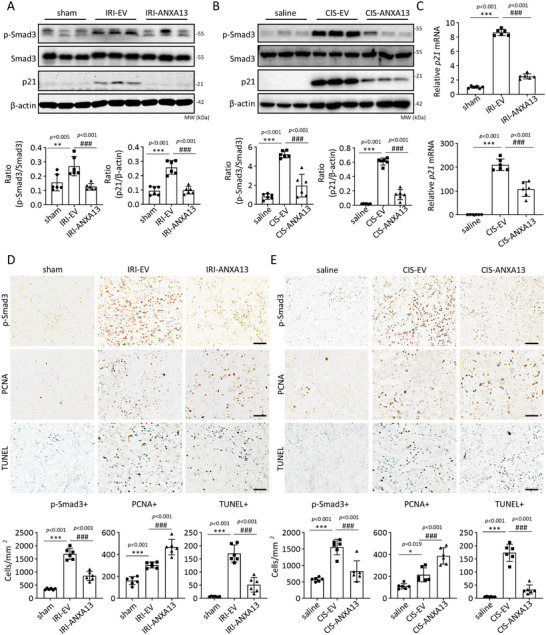
ANXA13 overexpression protects against AKI by inhibiting the TGF‐β/Smad3‐p21 G1 cell cycle arrest pathway in vivo and in vitro. (A,B) Western blot analyses of p‐Smad3 and p21 in IRI‐ and cisplatin‐induced AKI mice. (C) Relative mRNA levels of p21 in IRI‐ and cisplatin‐induced AKI mice. (D,E) Immunohistochemistry of p‐Smad3, PCNA, and TUNEL in IRI‐ and cisplatin‐induced AKI mice. Positive cells are labeled brown, while nuclei are counterstained with methyl green in the TUNEL‐stained sections. Data are reported as mean ± SD from groups of 6 mice in vivo. Statistical analysis was performed using one‐way ANOVA with Tukey's test for panels A, C (p21), D (PCNA), and F (PCNA), and one‐way ANOVA with Dunnett's test for panels B, C (KIM‐1), D (p‐Smad3 and TUNEL), and F (p‐Smad3 and TUNEL). ^**^ *p* <0.01, ^***^ *p* <0.001 versus sham or saline, ^##^ *p* <0.01, ^###^ *p* <0.001 versus IRI‐EV or CIS‐EV, scale bars = 50 µm.

To explore the potential regulatory mechanism of ANXA13 in AKI, RNA sequencing was performed on EV‐IRI‐AKI and ANXA13‐IRI‐AKI mice. A total of 921 differentially expressed genes (DEGs) were identified, of which 564 were downregulated (blue) and 357 were upregulated (red) after ANXA13 overexpression in IRI‐AKI mice (Figure 5A). Gene set enrichment analysis (GSEA) analysis showed that the cell cycle checkpoint and G1‐S specific transcription were significantly enriched, with their related genes regulated by ANXA13 (Figure 5B). Gene Ontology (GO) biological process (BP) enrichment analysis was conducted for the downregulated and upregulated DEGs. The BP enrichment results showed that the cellular response to transforming growth factor beta stimulus was highly enriched in the downregulated DEGs in ANXA13‐IRI‐AKI kidneys, whereas the upregulated DEGs were significantly enriched in the mitotic cell cycle (Figure 5C). As Smad3 can directly interact with the promoter of cyclin‐dependent kinase inhibitors (p21/p27) to cause TEC death during AKI via the G1 cell cycle arrest mechanism [18, 20, 21, 22], we further explored whether ANXA13 protects against AKI by inhibiting the TGF‐β/Smad3‐p21‐dependent cell cycle arrest mechanism. As shown in Figure 5D,E, Anxa13 overexpression inhibited TGF‐β1‐induced Smad3 activation and p21 dependent cell cycle arrest in the G1 phase in vitro. This was further confirmed in vivo, as Anxa13 overexpression in IRI‐ and cisplatin‐induced AKI mice significantly inhibited the phosphorylation of Smad3 (p‐Smad3) and upregulation of p21. This promotes TEC proliferation by highly expressing the PCNA antigen while inhibiting cell apoptosis (Figure 6).

**FIGURE 7:**
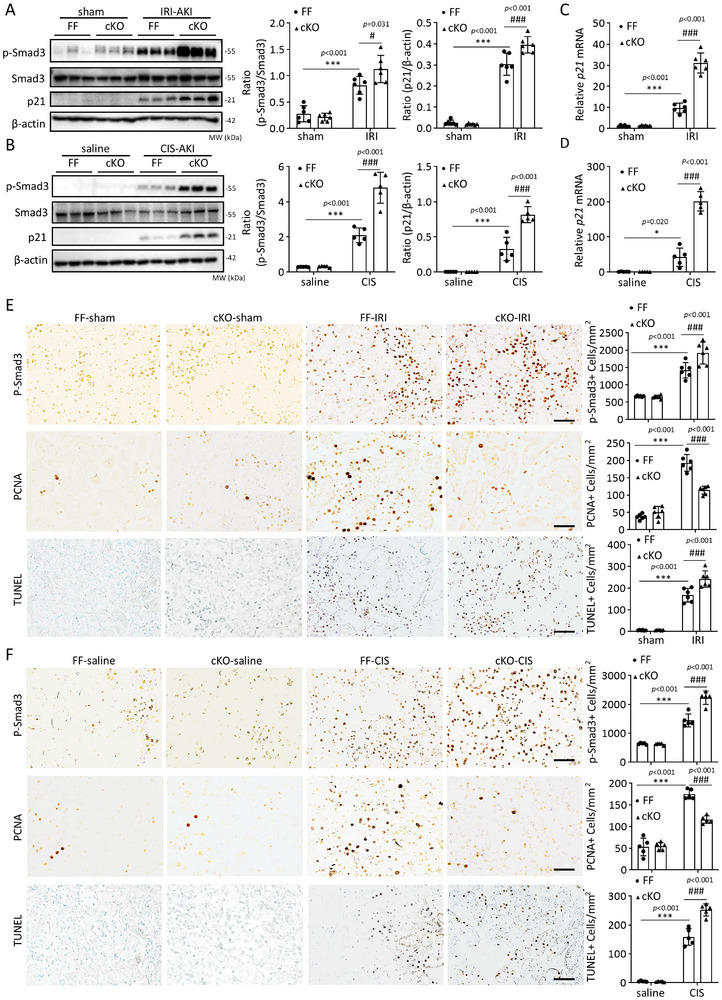
Deletion of *Anxa13* from renal tubular epithelial cells enhances activation of the TGF‐β/Smad3‐p21 G1 cell cycle arrest pathway in IRI‐ and cisplatin‐induced AKI mice. (A,B) Western blot analyses of p‐Smad3 and p21 in *Anxa13* ^flox/flox^ (FF) and *Anxa13* ^flox/flox^/*Ggt1*‐Cre (cKO) mice treated with IRI or cisplatin (CIS). (C,D) Relative mRNA levels of p21 in IRI or cisplatin‐induced AKI in *Anxa13* FF and cKO mice. (E,F) Immunohistochemical staining of p‐Smad3, PCNA, and TUNEL in IRI‐ or cisplatin‐induced AKI in *Anxa1*3 FF and cKO mice. Positive cells are labeled as brown, while nuclei are counter‐stained with methyl green in the TUNEL‐stained sections. Data are reported the mean ± SD from groups of 5–6 mice. Statistical analysis was performed using two‐way ANOVA with Tukey's test for panels A–F. ^***^ *p* <0.001 versus FF sham or saline; ^##^ *p* <0.001, ^###^ *p* <0.001 versus FF IRI or FF CIS. Scale bars = 50 µm.

In contrast, cKO of *Anxa13 f*rom renal tubules largely promoted the activation of Smad3 (p‐Smad3) and increased the p21 levels, resulting in increased cell apoptosis and inhibiting TEC proliferation by reducing the PCNA antigen (Figure 7). Thus, ANXA13 may protect against AKI by inhibiting the TGF‐β/Smad3‐p21 cell cycle arrest pathway.

**FIGURE 8:**
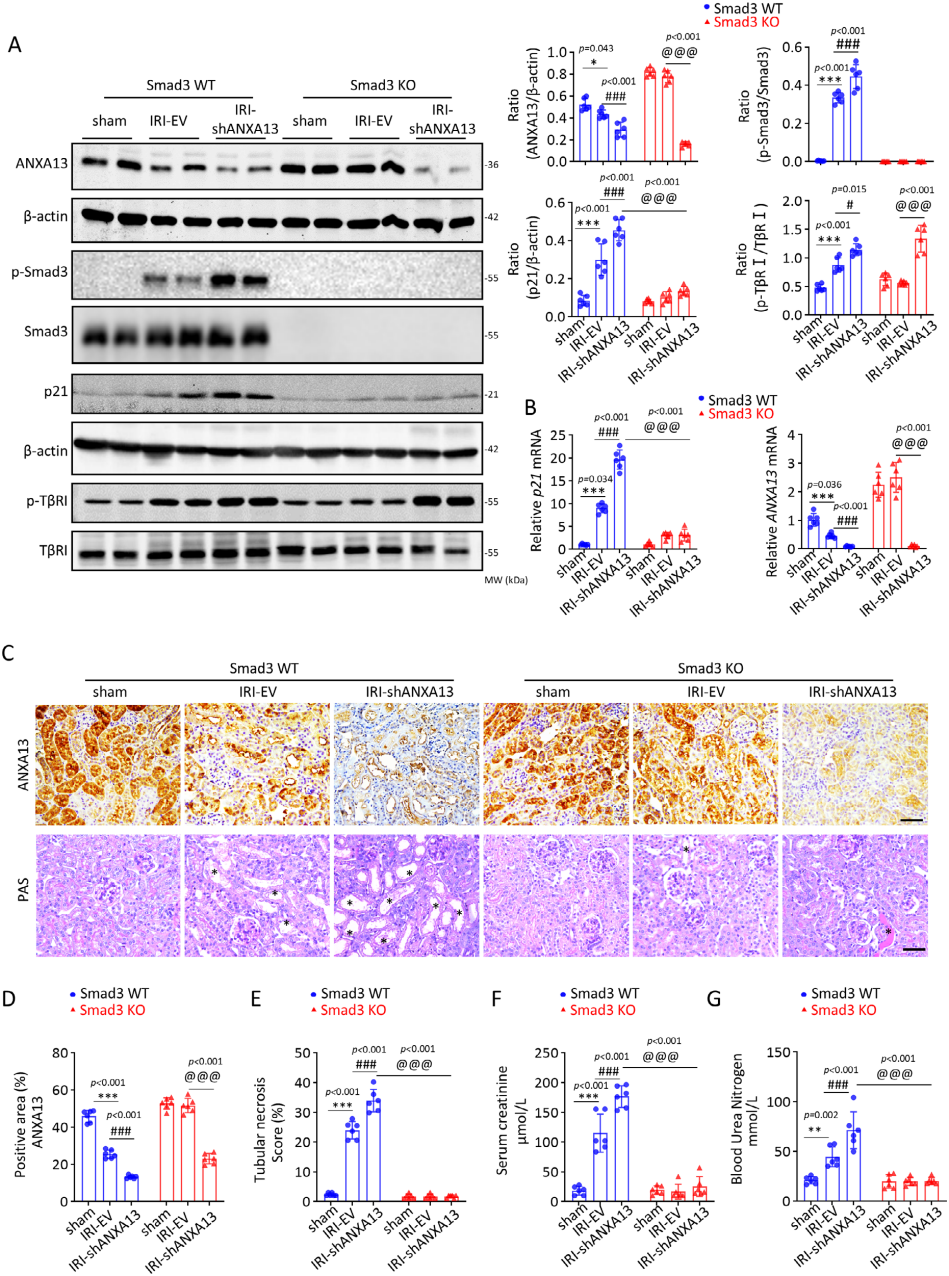
ANXA13 protects against AKI via Smad3‐dependent mechanisms. (A) Western blot analyses of ANXA13, p‐Smad3, p21, and p‐TβRI in IRI‐induced AKI in *Smad3* WT and KO mice. (B) Relative mRNA levels of ANXA13 and p21 in IRI‐induced AKI in *Smad3* WT and KO mice. (C) Immunohistochemical staining of ANXA13 and PAS staining for the detection of tubular necrosis (*) in IRI‐induced AKI in *Smad3* WT and KO mice. (D–E) Quantitative analysis of renal ANXA13 and tubular necrosis. (F–G) SCr and BUN levels in IRI‐induced AKI in *Smad3* WT and KO mice. Data are reported the mean ± SD from groups of 6 mice. Statistical analysis was performed using two‐way ANOVA with Tukey's test for panels A–G. ^*^ *p* <0.05, ^**^ *p* <0.01, ^***^ *p* <0.001 versus *Smad3* WT sham, ^#^ *p* <0.05, ^###^ *p*<0.001 versus *Smad3* WT IRI‐EV, ^@@@^ *p*<0.001 versus *Smad3* KO IRI shANXA13.

To further confirm the mechanism by which ANXA13 protects against AKI via Smad3, we performed studies in AKI mice induced in *Smad3* wild‐type (WT) and *Smad3* knockout (KO) mice by kidney‐specific silencing of ANXA13 using ultrasound microbubble‐mediated shANXA13 plasmids. In Smad3 WT mice, silencing renal ANXA13 largely promoted IRI‐induced TGF‐β/Smad3 signaling (p‐TβRI and p‐Smad3) and p21 expression, resulting in worsening IRI‐induced AKI by largely elevating SCr and BUN levels and inducing tubular necrosis (Figure 8). In contrast, mice lacking Smad3 were protected from IRI‐induced AKI and, more importantly, were able to prevent shANXA13‐induced severe AKI by abolishing the p21‐mediated G1 cell cycle arrest pathway (Figure 8). These observations strongly suggest that ANXA13 protects against AKI by targeting the TGF‐β/Smad3 signaling pathway.

### 2.7 ANXA13 Inactivates TGF‐β/Smad3 Signaling by Directly Interacting with TβRI

**FIGURE 9:**
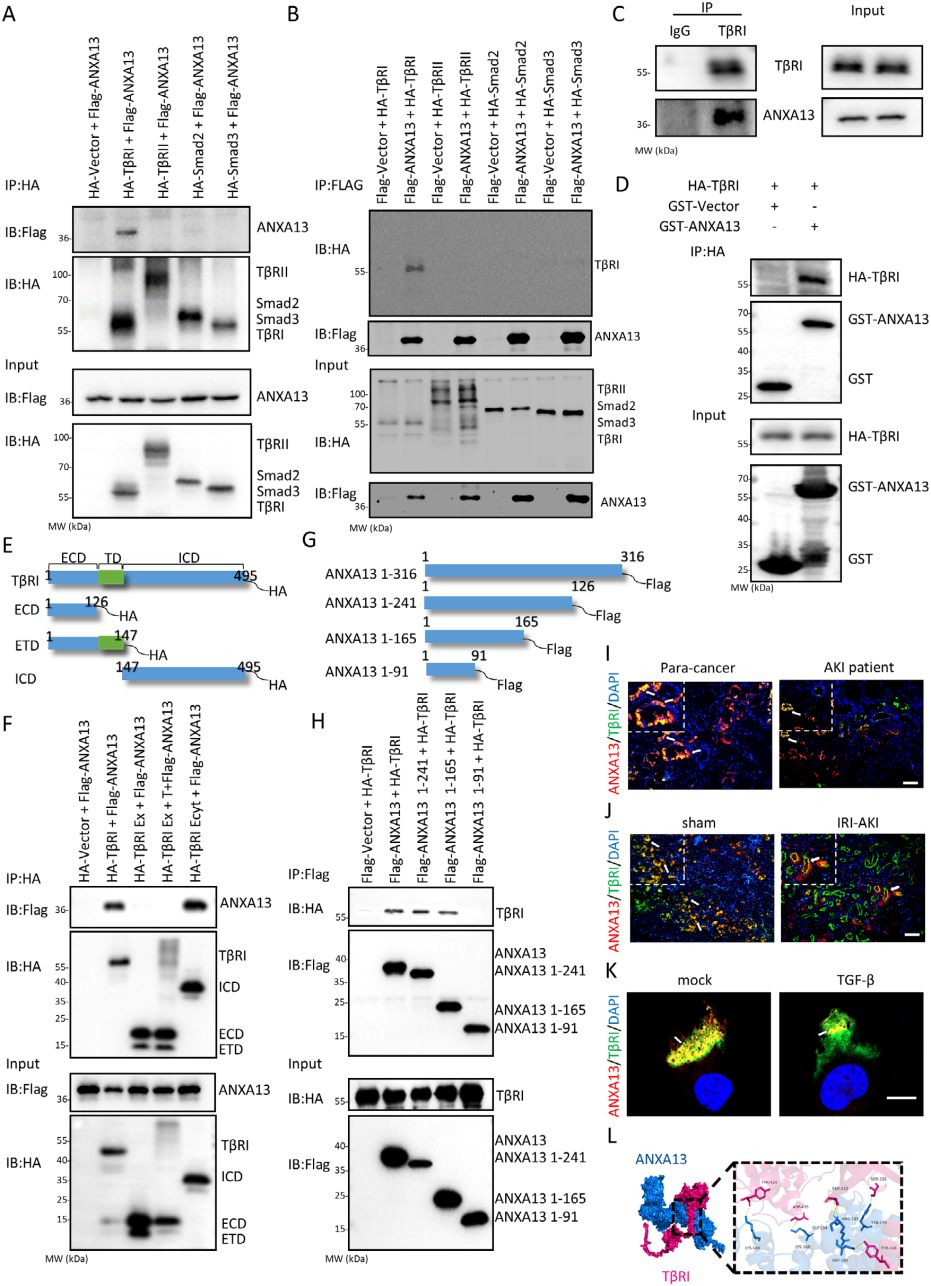
ANXA13 directly interacts with TβRI. (A,B) Interaction between ANXA13 and TβRI assayed using Co‐IP in HEK293T cells co‐expressing Flag‐ANXA13 and HA‐tagged TβRI, TβRII, Smad2, and Smad3. (C) Endogenous interaction between ANXA13 and TβRI assayed using Co‐IP in HK‐2 cells. (D) Interaction between ANXA13 and TβRI assayed by GST pulldown in vitro. (E) Schematic representation of full‐length and truncated TβRI proteins. (F) Interaction between ANXA13 and functional domains of TβRI, including amino acids 1–126 (ECD: extracellular domain), 1–147 (ETD: extracellular and transmembrane domain), 147–495 (ICD: intracellular domain), as determined using Co‐IP in HEK293T cells. (G) Schematic representation of full‐length and truncated ANXA13. (H) Interaction between TβRI and truncated ANXA13 protein. (I–K) Two‐color immunofluorescence shows that ANXA13 (red) co‐localizes with TβRI(green) in the kidneys of patients (I), mice (J), and HK‐2 cells (K). (L) Molecular docking analysis of TβRI (pink) and ANXA13 (blue). White arrows indicate the co‐localization of ANXA13 (red) and TβRI(green). Scale bars = 50 µm.

An interesting finding of this study was that silencing renal ANXA13 with shANXA13 caused more severe AKI in Smad3 WT mice by largely enhancing TGF‐β/Smad3 signaling with higher levels of phosphorylated TGF‐β receptor type 1 (p‐TβRI) and p‐Smad3, which was prevented in *Smad3* KO mice without altering high levels of p‐ TβRI (Figure 8A). This suggests that ANXA13 may interact with TβRI to inactivate the downstream Smad3 signaling. This was examined by co‐transfecting HEK293T cells with Flag‐ANXA13 and HA‐TβRI/ HA‐TβRII/ HA‐Smad2/HA‐Smad3. Co‐immunoprecipitation assays (Co‐IP) revealed that Flag‐ANXA13 protein directly binds to HA‐TβRI, but not to other HA‐tagged proteins such as HA‐TβRII/ HA‐Smad2/HA‐Smad3, to form a complex (Figure 9A,B). The endogenous Co‐IP experiment further supported the notion that endogenous ANXA13 is directly associated with TβRI (Figure 9C). GST pulldown analysis showed an in vitro interaction between GST‐ANXA13 and HA‐TβRI (Figure 9D). To further confirm the interaction domain of ANXA13 and TβRI, we constructed the Flag‐ANXA13 and HA‐TβRI truncated protein expression plasmids. The structural data from the UniProt Database also revealed that TβRI consists of an extracellular domain, a transmembrane domain, and an intracellular domain with a phosphorylation domain and a protein kinase domain (Figure 9E). To clarify which domain of ANXA13 interacts with TβRI, Co‐IP was performed to detect the interaction between ANXA13 and different domains of TβRI. As shown in Figure 9F, our results revealed that ANXA13 interacts with the intracellular domain of TβRI. Additionally, the interaction between the truncated ANXA13 protein and TβRI was also detected and showed that TβRI was able to interact with the full length ANXA13, ANXA13 (1–241) and ANXA13 (1–165), but failed to interact with the ANXA13 (1–91) (Figure 9H). Consistent with the Co‐IP result, double‐immunofluorescence also revealed the colocalization of ANXA13 and TβRI in vitro and in vivo (Figure 9I–K; Figure S8). Furthermore, molecular docking analysis predicted that ANXA13 LYS‐149, LYS‐168, GLY‐184, ARG‐185, ASP‐180 and TYR‐179 could form hydrogen bonds with TβRI TYR‐424, ASP‐435, SER‐210, SER‐235, and TYR‐146, respectively (Figure 9L). Together, these findings suggest a specific interaction between ANXA13 and TβRI.

**FIGURE 10:**
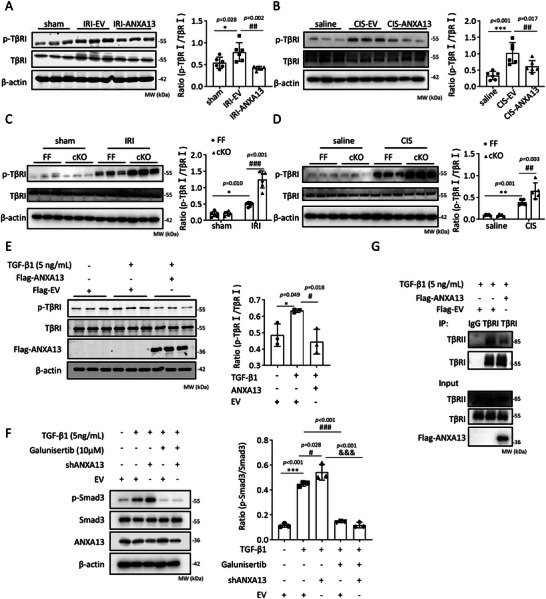
ANXA13 inhibits the phosphorylation of TβRI. (A,B) Western blot analysis of the phosphorylation levels of TβRI (p‐TβRI) in IRI‐ and cisplatin‐induced AKI (C,D) Western blot analysis of p‐TβRI in IRI‐ and cisplatin‐induced AKI in *Anxa1*3^lox/flox^ (FF) and *Anxa1*3^flox/flox^ /*Ggt1*‐Cre (cKO) mice. (E) Western blot analysis of p‐TβRI in HK‐2 cells treated with TGF‐β1. (F) Western blot analysis of the phosphorylation levels of Smad3 (p‐Smad3) in the HK‐2 cells. (G) Co‐IP analysis showed that ANXA13 overexpression inhibited the binding of TβRII and TβRI. Data are reported as the mean ± SD from group of 5–6 mice in vivo or 3 independent experiments in vitro. Statistical analysis was performed using one‐way ANOVA with Tukey's test for A, B, E, and F, and two‐way ANOVA with Tukey's test for C and D. ^*^p <0.05, ^**^ *p* <0.01, ^***^ *p* <0.001 versus Flag‐EV or sham or Saline or FF sham or FF saline; ^#^ *p* <0.05, ^##^ *p* <0.01, ^###^ *p* <0.001 versus Flag‐EV with TGF‐β1 or IRI‐EV or CIS‐EV or FF IRI or FF CIS; ^&&&^ *p* <0.001 versus TGF‐β1+shANXA13.

Functionally, we detected that Anxa13 overexpression significantly attenuated the phosphorylation of TβRI in the AKI kidney (Figure 10A,B), whereas genetic deletion of Anxa13 from renal tubules notably enhanced TβRI phosphorylation (Figure 10C,D). Consistent with these in vivo findings, Anxa13 overexpression in HK‐2 cells suppressed TGF‐β1‐induced phosphorylation of TβRI (Figure 10E). Furthermore, using a selective TβRI kinase inhibitor (galunisertib), which exerts its inhibitory effect on TGF‐β/Smad signaling by occupying the ATP‐binding site of TβRI and consequently reducing p‐Smad3 levels [23], we demonstrated that silencing Anxa13 largely enhanced TGF‐β1 induced phosphorylation of Smad3 in HK‐2 cells, which was blocked by galunisertib treatment (Figure 10F). No significant differences in p‐Smad3 levels were observed in HK‐2 cells treated with galunisertib or galunisertib+shANXA13(Figure 10F). These results indicate that ANXA13 may interact with TβRI to exert its inhibitory effect on TGF‐β/Smad3 signaling. As binding to TβRII is a prerequisite for TβRI phosphorylation [24], we investigated whether ANXA13 modulates the formation of this receptor complex. Co‐IP results demonstrated that ANXA13 overexpression inhibited the binding of TβRII and TβRI (Figure 10G). These findings suggest that ANXA13 may inhibit TGF‐β/Smad3 signaling through direct interaction with TβRI, and disruption of TβRII/I complex formation, thereby preventing TβRI phosphorylation.

### 2.8 Smad3 Binds to ANXA13 3’UTR to Negatively Regulate the Transcription of ANXA13

**FIGURE 11:**
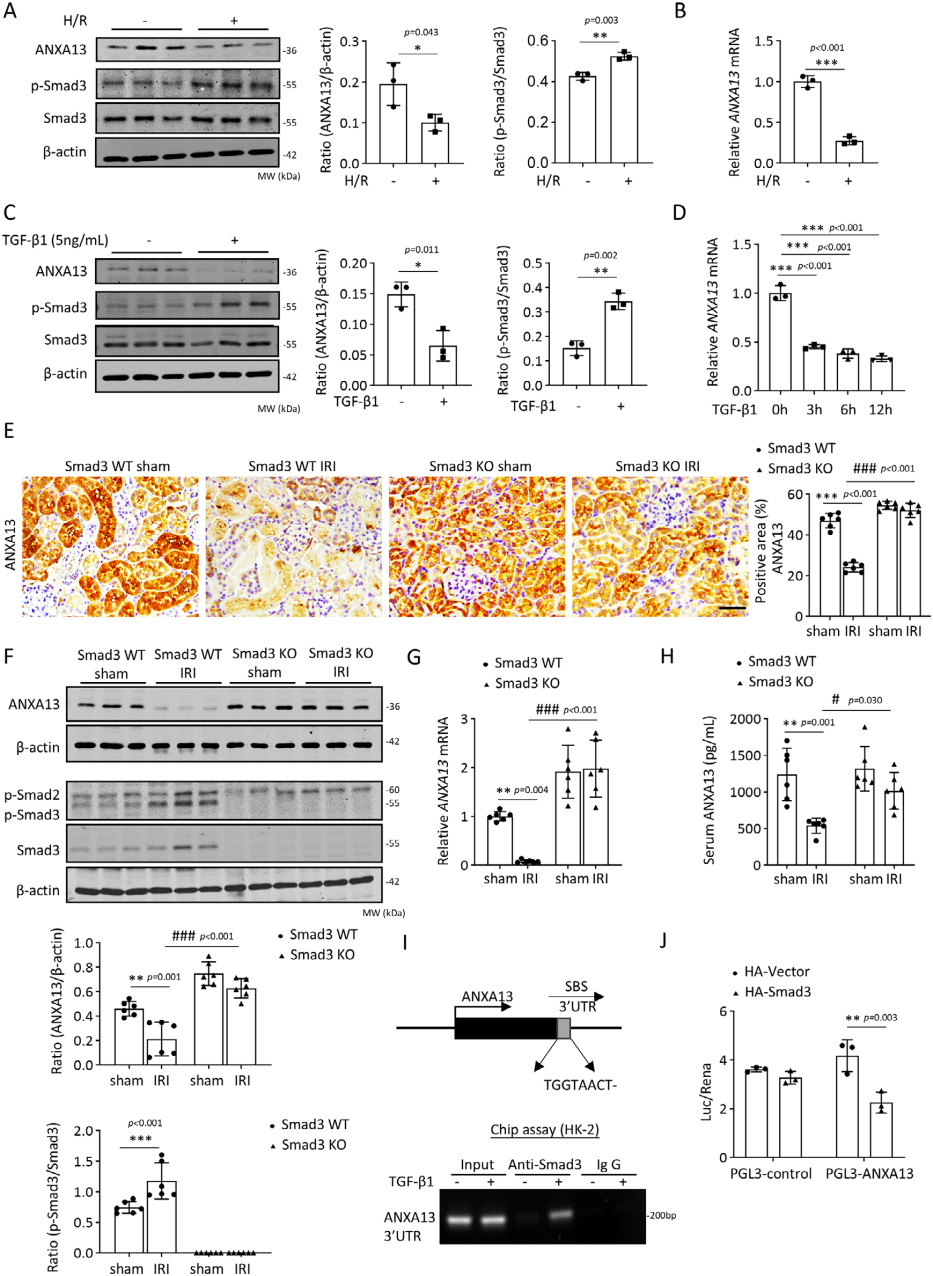
ANXA13 is downregulated by IRI and TGF‐1 via a Smad3‐dependent mechanism in vivo and in vitro. (A) Western blot analysis of ANXA13 and p‐Smad3 in HK‐2 cells treated with H/R. (B) Relative mRNA levels of ANXA13 in HK‐2 cells treated with H/R. (C) Western blot analyses of ANXA13 and p‐Smad3 in HK‐2 cells treated with TGF‐β1 (5 ng/mL) for 12h. (D) Relative mRNA levels of ANXA13 in HK‐2 cells treated with TGF‐β1 (5 ng/mL) at 0, 3, 6, 12 h. (E) Immunohistochemical staining of ANXA13 in IRI‐induced AKI in *Smad3* WT and KO mice. (F) Western blot analysis of ANXA13 and p‐Smad3 in IRI‐induced AKI in *Smad3* WT and KO mice. (G) Relative mRNA levels of ANXA13 in IRI‐induced AKI in *Smad3* WT and KO mice. (H) Serum ANXA13 levels in IRI‐induced AKI in *Smad3* WT and KO mice. (I) ChIP assay shows that binding of Smad3 to ANXA13 3ʹUTR region is greatly enhanced in HK‐2 cells in response to TGF‐β1 (5 ng/mL) in 3 h. (J) Luciferase activity detected via dual‐luciferase reporter assay. Data are reported as mean ± SD from groups of 6 mice in vivo or 3 independent experiments in vitro. Statistical analysis was performed using an unpaired t‐test for panles A–C, one‐way ANOVA with Tukey's test for D, and two‐way ANOVA with Tukey's test for panels F–H, J. ^*^ *p*<0.05, ^**^ *p* <0.01, ^***^ *p* <0.001 versus *Smad3* WT sham or control group without H/R or TGF‐β1 treatment or HA‐Smad3 + PGL3‐ANXA13; ^#^ *p* <0.05, ^##^ *p* <0.01, ^###^ *p* <0.001 versus *Smad3* KO IRI.

Next, we examined whether TGF‐β/Smad3 signaling was also responsible for the loss of renal ANXA13 in the AKI kidneys by culturing HK‐2 cells with TGF‐β1 or hypoxia/reoxygenation (H/R). As shown in Figure 11A–D, H/R or TGF‐β1 treatment significantly inhibited ANXA13 expression at both the mRNA and protein levels. This was observed in vivo, where IRI‐induced AKI in Smad3 WT mice was associated with the loss of ANXA13, which was prevented in mice lacking Smad3 (Figure 11E–H). To clarify the mechanism by which Smad3 inhibits ANXA13 expression, a Smad3‐binding site was predicted in the 3’UTR region of ANXA13 using the Gene Transcription Regulation Database (GTRD). The ChIP assay further indicated that Smad3 binds to the 3’UTR of ANXA13 in HK‐2, which was enhanced by TGF‐β1 treatment (Figure 11I). The luciferase activity assay showed that Smad3 overexpression decreased the luciferase activity of ANXA13 3’UTR, suggesting that Smad3 regulates ANXA13 expression (Figure 11J). The regulatory role of Smad3 in renal ANXA13 expression was also demonstrated in AKI mice, in which mice lacking Smad3 were protected from IRI‐induced loss of renal ANXA13 and the development of AKI (Figure 8C–F). Thus, ANXA13 is negatively regulated by TGF‐β/Smad3 signaling and in turn protects against AKI by inhibiting TGF‐β/Smad3‐p21‐dependent G1 cell cycle arrest.

## 3 Discussion

Cell injury and TEC death are prominent pathological features of AKI [25]. Consequently, therapeutic strategies aimed at either preventing TEC death or promoting the repair of injured TECs are critical approaches for AKI treatment. A novel and significant finding of this study is the identification of the protective role of ANXA13 in AKI. This was demonstrated by the findings that overexpressing renal Anxa13 protected against IRI‐ and cisplatin‐induced AKI, whereas silencing renal Anxa13 resulted in worsening both IRI‐ and cisplatin‐induced AKI. These effects were further substantiated in mice with cKO of renal tubular Anxa13, where the specific deletion of tubular Anxa13 led to more severe AKI, as indicated by significantly increased levels of SCr, BUN, KIM‐1, and tubular necrosis. Thus, ANXA13 is a previously unrecognized protein that protects the kidneys from AKI.

Accumulating evidence has demonstrated that ANXAs may be potential biomarkers for cancer progression, including in ovarian cancer and acute myeloid leukemia [26, 27], and are associated with the clinical outcomes in patients with renal cell carcinoma (RCC) [28]. In the current study, we observed that ANXA13 was highly expressed in the normal kidney, presumably in proximal tubular epithelial cells; however, this expression was lost when AKI developed in both patients with AKI and animal models. The loss of renal TEC ANXA13 was significantly associated with decreased serum ANXA13 but increased urinary ANXA13, which was correlated with the onset and severity of AKI. Furthermore, changes in ANXA13 may predict AKI regression as patients recovered from AKI were characterized by increased serum and decreased urinary ANXA13 levels clinically. Proximal TECs are the major victims of AKI [29]. Considering the abundance of ANXA13 expressed in proximal tubular cells, tubular injury may trigger the rapid release of ANXA13 into the urinary space, resulting in elevated urinary levels of ANXA13. Concurrently, tubular cell injury may lead to reduced ANXA13 synthesis and secretion, thereby contributing to the observed decrease in serum ANXA13 levels in AKI. In this regard, changes in serum and urinary ANXA13 levels could reflect the TEC injury in AKI and may be a new potential biomarker for AKI. However, the sample size of the current study was relatively small. Further clinical trials with large‐scale clinical samples are warranted to identify ANXA13 as a novel biomarker for AKI, including in patients with progressive AKI to comprehensively evaluate the clinical utility and comparative advantages of ANXA13 over currently available biomarkers.

Mechanistically, we observed that ANXA13 bound directly to the TβRI, but not to TβRII, Smad2, or Smad3, to exert its inhibitory effect on Smad3‐p21‐mediated cell death during AKI. Emerging evidence suggests that cell cycle arrest is a key mechanism driving AKI outcomes (progression/recovery) and is a source of predictive biomarkers for moderate‐to‐severe cases [22, 30]. Smad3 is a key mediator of AKI as Smad3 can directly bind to the promoters of the cyclin‐dependent kinase inhibitor proteins p21/p27, leading to cell death through G1 cell cycle arrest [18, 20, 31, 32]. We previously reported that renal p21 is increased in kidneys with AKI, and genetic deletion or pharmacological inhibition of *Smad3* can block p21‐mediated TEC death during AKI [33, 34], which is consistent with current findings in present study. Furthermore, we discovered that ANXA13 could directly interact with the intracellular domain of TβRI which contains GS domain and suppressed the phosphorylation of the TβRI. After TGF‐β1 binds to the TβRII, the recruitment of TβRI and the interaction between TβRII and TβRI are essential for the phosphorylation of the GS domain of TβRI, which enables TβRI to activate downstream Smad3 signaling [24]. Thus, we performed the Co‐IP experiment to detect whether ANXA13 modulates the TβRII/I complex formation and demonstrated that overexpression of ANXA13 did inhibit the binding between TβRII and TβRI. Although the ANXAs are not a class of phosphatases, accumulated evidence also revealed that ANXAs are not a class of phosphatases, accumulating evidence has revealed that ANXAs can regulate phosphorylation via protein‐protein interactions. Xu et al. reported that ANXA5 inhibits the phosphorylation of pyruvate kinase M2 (PKM2) Y105 by binding to PKM2 ASP101, LEU104 and ARG106 [35], whereas ANXA7 promotes T‐cell intracellular antigen‐1 (TIA1) phosphorylation by interacting with TIA1 [36]. Therefore, ANXA13 may reduce TβRI phosphorylation by interacting with the intracellular domain of TβRI, thereby inhibiting the TβRII/I complex formation. To determine whether the inhibitory effect of ANXA13 on TGF‐β/Smad3 signaling pathway is TβRI dependent or not, we blocked TβRI by using a TβRI specific inhibitor, galunisertib. Notably, pharmacological inhibition of TβRI completely attenuated the shANXA13‐induced upregulation of p‐Smad3 levels. Collectively, these findings revealed a protective role and detailed mechanism for ANXA13 in negatively regulating TGF‐β/Smad3 signaling.

Intrinsically, we also found that the activation of Smad3 could, in turn, suppress the ANXA13 expression. This finding aligns with the observation that the activation of Smad3 was associated with the loss of renal ANXA13 in the IRI‐AKI kidneys. Further studies identified a Smad3 binding site in the 3’UTR of human ANXA13, and this interaction was enhanced in response to TGF‐β1 as demonstrated using ChIP assay and luciferase activity assays. Functionally, the deletion of *Smad3* protected against IRI‐induced AKI by preventing the loss of renal TEC ANXA13, suggesting a negatively regulatory role of Smad3 in renal ANXA13 expression during AKI. It is likely that, during AKI, activation of TGF‐β/Smad3 signaling downregulated renal ANXA13 expression, which in turn, sustains TGF‐β/Smad3 signaling by weakening the inhibitory effect of ANXA13 on TβRI phosphorylation. This may also well explain the loss of ANXA13 in the AKI kidney with overactive TGF‐β/Smad3 signaling and suggests that the TGF‐β/Smad3‐ANXA13 circuit is a key regulatory mechanism during the injury and recovery processes of AKI.

Owing to the incomplete characterization of ANXA13 functional domains, at present we could not identify the specific function of ANXA13 domains that mediate their binding to the TβRI intracellular region. Furthermore, as the TGF‐β/Smad3 pathway is a key pathway and therapeutic target for AKI [22, 37], compounds targeting smad3, such as SIS3 and flavonoids, have shown efficacy in attenuating renal injury in experimental AKI models [22, 34, 37]. However, these compounds have not yet been applied in the clinic due to potential nephrotoxicity and insufficient clinical evidence [38]. Our finding that ANXA13 is a TEC‐expressed negative regulator of TGF‐β/Smad3 signaling provides critical mechanistic complementarity by revealing an endogenous regulatory mechanism distinct from extraneous pharmacological inhibitors. This suggests that ANXA13 supplementation may offer a safe and physiologically aligned therapeutic strategy for AKI, which warrants further clinical investigations.

## 4 Conclusion

In conclusion, ANXA13 is downregulated during AKI under the negative regulation of TGF‐β/Smad3 signaling. Functionally, ANXA13 confers renal protection in AKI and exerts its protective effect by directly binding to TβRI and inhibiting its phosphorylation, thereby suppressing the TGF‐β/Smad3‐p21‐mediated cell cycle arrest pathway. Thus, ANXA13 may represent a novel therapeutic agent for AKI.

## 5 Experimental Section

### 5.1 Clinical Specimens

Five kidney specimens for immunohistochemistry (IHC) were collected from patients diagnosed with AKI at The Third Affiliated Hospital of Southern Medical University. Five para‐cancer normal kidney tissues and five renal biopsies with minimal changes in disease were collected as control samples. Moreover, 44 serum and 40 urine samples were collected from patients with AKI at the same hospital. Additionally, 31 and 27 urine samples from healthy volunteers were used as controls.

AKI was diagnosed according to the *Kidney* *Disease* *Improving* *Global* *Outcomes* (KDIGO) guidelines. According to the consensus report of the Acute Dialysis Quality Initiative (ADQI) 16 Workgroup, no standardized definition of recovery from AKI exists, as different studies have established their own recovery criteria based on different research objectives and clinical contexts [39]. Therefore, we defined renal recovery as a ≥33% decrease from AKI SCr or a return to SCr values ≤150% of the baseline as previously reported [40, 41].

The clinical characteristics of the patients with AKI involved in this study are listed in Table S1–S4. Written informed consent was obtained from each participant, and the study was approved by the Ethics Committee of Southern Medical University (2023‐059).

### 5.2 Generation of *Anxa13* cKO Mice and *Smad3* Knockout (KO) Mice

*Smad3* KO C57BL/6 mice were provided by Dr. C. Deng [42]. *Anxa13* ^flox/flox^ (FF) mice were constructed via Cre‐loxP‐mediated recombination in mice (Figure S6) and were purchased from Cyagen Biosciences. To generate *Anxa13* ^flox/flox^/*Ggt1*‐cre (cKO) mice, *Anxa13* FF mice were mated with tubule‐Cre (*Ggt1*‐cre) mice purchased from Cyagen Biosciences. Littermates male *Anxa13* FF mice were used as control. *Anxa13* FF or *Anxa13* cKO mice were identified via genotyping with primers (Table S5), and Smad3 KO mice were determined as previously described [20].

### 5.3 Mouse Models of AKI

All animal experiments were conducted in male C57BL/6J mice (8–10 weeks) following the protocol approved by the Animal Ethics Experimentation Committee of the Chinese University of Hong Kong (20‐243‐MIS). For the cisplatin‐induced AKI model, mice were intra peritoneally injected with an optimal dose of cisplatin at 15 mg/kg based on previous study [43]. All mice were sacrificed on day 3 after injection. For the IRI‐induced AKI model, the bilateral renal pedicles were clamped for 30min as previously described [20]. Briefly, the mice were anesthetized via an intraperitoneal injection of ketamine/xylazine (100mg/10mg per kg). Blood circulation in both kidneys was blocked by clamping the bilateral renal pedicles by using microvascular clampers for 30min, followed by the removal of the microvascular clampers to allow blood reperfusion. During the surgery, the body temperature of the mice was maintained at 37 °C using a homeothermic pad. The same procedure without renal pedicle clamping was performed on the sham control mice. Finally, the mice were sacrificed by overdose of anesthesia with 100mg/kg pentobarbitone at different experimental time points (days 1, 3 and 7), and the kidney tissue and blood were collected for examinations.

To study the regulatory role of Smad3 in ANXA13 expression, AKI was induced in *Smad3* KO/WT mice using IRI as described above. To confirm the necessary role of ANXA13 in Smad3‐mediated AKI, ANXA13 expression was silenced in the kidneys of *Smad3* KO/WT mice with AKI.

### 5.4 Ultrasound‐Microbubble‐Mediated Overexpression and Knockdown of Renal ANXA13 in the AKI kidney

ANXA13‐expressing or shRNA plasmids or empty vector controls at 200 µg/mouse were mixed with lipid microbubbles (SonoVue; Bracco, Milan, Italy) at a ratio of 1:1. A 400 µL aliquot of the mixture was injected into the mice via the tail vein. Immediately after the plasmid‐bearing microbubbles injection, the mice were treated with a pulse‐wave output of 1 mHz at 2 W/cm2 for a total of 10 min by rotating to each side at 30s intervals, as previously described [20]. The principle of ultrasound‐microbubble‐mediated gene transfer is based on the use of ultrasound contrast agents to lower the threshold for cavitation triggered by ultrasound energy and to incorporate the genes of interest into gene‐bearing microbubbles. Once these DNA‐bearing microbubbles are injected intravenously, and ultrasound energy can be applied to the target region or organ. As the gene‐bearing microbubbles enter the insonation region, cavitation results in the rapid release of genes from the microbubbles. Cavitation may cause a local shockwave that increases cell permeability, effectively improving the cellular uptake of DNA while bypassing lysosomal degradation [44]. Through this method, the expressing plasmids or genes can be transfected into the kidney without genome integration risks, as determined by FITC‐ODN, Flag‐proteins, or miRNAs/lncRNAs as previously describe [20, 22, 45, 46, 47, 48]. One day after overexpressing or knocking down Anxa13, groups of 6 mice were induced with IRI or cisplatin, as described above.

To evaluate the efficiency and tissue specificity of ultrasound‐microbubble‐mediated gene transfer into the kidney, the GFP‐expressing plasmid or empty vector at dosages of 100, 200, 300µg per mouse was mixed with microbubbles and immediately injected into normal mice via the tail vein, followed by ultrasound exposure as described above. The mice were sacrificed on day 2 and GFP expression in the ultrasound‐treated kidney and other organs without ultrasound exposure, including the liver, heart, and lung, were accessed by immunofluorescence. Consistent with our previous results [20, 49], GFP expression was largely detected in the ultrasound‐treated kidney in a dose‐dependent manner, peaking at a dose of 200 µg, in which most of intrinsic kidney cells (largely tubular epithelial cells) were highly expressed GFP signals, whereas only a few or undetectable GFP‐expressing cells were observed in the liver, heart and lung without ultrasound treatment (Figure S4A,B).

Furthermore, to assess the safety of the ultrasound microbubble‐mediated ANXA13 delivery method, serum creatinine, lactate dehydrogenase (LDH), alanine aminotransferase (ALT), and aspartate aminotransferase (AST) levels were determined in normal control mice and those treated with the ultrasound microbubble‐mediated empty vector or ANXA13‐expressing plasmids using commercial assay kits, according to the manufacturer's instructions (Nanjing Jiancheng, China). Histological analysis was conducted in the kidneys, livers, hearts, and lungs treated with or without an ultrasound‐microbubble‐mediated empty vector or ANXA13‐expressing plasmids using hematoxylin and eosin (H&E) staining (Solaibao, China). The results showed no significant differences in SCr, LDH, ALT, or AST levels among the normal control, empty vector control, and ANXA13‐treated groups (Figure S4C). H&E staining revealed no detectable tissue injury in any of the examined organs with or without ultrasound microbubble treatment (Figure S4D). These results confirmed the safety of this gene transfer technique.

### 5.5 Renal Function and Histology

Blood samples were collected by cardiac puncture from anaesthetized mice, and the serum was subsequently extracted for analysis. SCr levels were measured using the Creatinine LiquiColor Test (Stanbio Laboratory, Texas, USA), and BUN levels were measured using a Urea Assay Kit (Nanjing Jiancheng, China). Kidney tissues were fixed with 4% paraformaldehyde, embedded in paraffin and sectioned (3 µm) for Periodic Acid‐Schiff (PAS) staining. A total of 500 tubules were examined, and the percentage of necrotic tubules was calculated.

### 5.6 Cell Culture

Human renal tubular epithelial cell lines (HK‐2 cells) and human embryonic kidney (HEK293T) cells were purchased from the American Type Culture Collection (ATCC) (Manassas, VA, USA). HEK293T cells were cultured in DMEM supplemented with 10% FBS and 1% penicillin and streptomycin (Gibco, USA). HK‐2 cells were cultured in DMEM‐F12 medium supplemented with 10% FBS and 1% penicillin and streptomycin (Gibco, USA). Cells were stimulated with or without TGF‐β1 (5 ng/mL, Peprotech 100‐21C) at different time points. For the induction of H/R injury, HK‐2 cells were cultured in the AnaeroPack system (Mitsubishi Gas Chemical, Japan) with oxygen levels less than 0.1% at 37 °C for 24 h followed by 6 h of reoxygenation.

### 5.7 Co‐Immunoprecipitation Assays (Co‐IP)

Protein expressing plasmids were used in this study including Flag‐ANXA13, Flag‐ANXA13 1–241, Flag‐ANXA13 1–165, Flag‐ANXA13 1–91, HA‐TβRI, HA‐TβRII, HA‐Smad2, HA‐Smad3, HA‐TβRI‐ECD (extracellular domain), HA‐TβRI‐ETD (extracellular and transmembrane domain), HA‐TβRI‐ICD (intracellular domain). For exogenous IP, HEK293T cells were transfected with various plasmid combinations. For endogenous IP, HK‐2 cells were used. Proteins were extracted with cell lysis buffer for Western and IP (P0013, Beyotime, China) and 500µg proteins was immunoprecipitated with Dynabeads Protein G (1007D, Invitrogen, USA) or anti‐HA Nanobody Magarose Beads (KTSM1335, AlpalifeBio, Shenzhen, China) according to the manufacturer's instructions. After washing, the immunoprecipitates were boiled in SDS loading buffer for 5min for western blot analysis. The primers used to construct protein expressing plasmids in this study are listed in Table S5.

### 5.8 GST Pulldown Assay

GST pulldown assay was conducted as previously described [50]. Briefly, GST vector control and GST‐tagged ANXA13 were transformed into *Escherichia coli* BL21 (CD901‐03, TransGen Biotech, China) and cultured at 37 °C for 2h. Protein expression in the bacteria was induced with 1 mM IPTG at 16°C overnight. GST proteins were purified using glutathione beads according to the manufacturer's instruction (P2260s, Beyotime, China). Then 500 µg proteins from HEK293T cells transfected with HA‐ TβRI were extracted and incubated with GST proteins bound to glutathione beads at 4 °C for 4h. The GST beads were washed three times, boiled in loading buffer, and detected by western blot.

### 5.9 ChIP Assay

HK‐2 cells were treated with 5 ng/mL TGF‐β1 for 3h. ChIP assay was performed using a Simple ChIP Enzymatic Chromatin IP Kit (Cell Signaling 9003). An anti‐Smad3 (Cell Signaling Technology 9523, 1:50) and IgG (Cell Signaling Technology 3900) antibodies were used for immunoprecipitation. The primers were listed in Table S5.

### 5.10 Luciferase Reporter Assay

HEK293T cells seeded in 12‐well plates were transfected with 200 ng of ANXA13 3ʹUTR luciferase reporter plasmid or control plasmid (firefly luciferase) and 20 ng of pRL‐TK (Renilla luciferase plasmid), together with empty vector plasmid or HA‐Smad3 plasmid for 24 h. After transfection, luciferase activity was measured using the Dual‐Luciferase Reporter Assay System (Promega, San Luis Obispo, CA, USA) according to the manufacturer's protocol. Firefly luciferase activity was normalized to Renilla luciferase activity to determine the relative reporter activity.

### 5.11 TUNEL Assay

Renal cell apoptosis was detected using a TUNEL Assay Kit‐HRP‐DAB (ab‐206386, Abcam) according to the manufacturer's instructions. TUNEL‐positive nuclei were identified using a Leica CRT6000 Light Microscope and expressed as cells/mm^2^.

### 5.12 Real‐Time PCR

Total RNA was extracted from the cells and kidney tissues using TRIzol reagent (Invitrogen) according to the manufacturer's protocol. RNA was then converted to cDNA using the HisScript II Reverse Transcription Kit (Novizan Biotechnology Co., Ltd., Nanjing, China). The PCR program for reverse transcription was as follows: 50 °C for 15 min and 85 °C for 5 s. mRNA expression was detected using a QuantStudio 7 Flex and SYBR Green Supermix (Bio‐Rad). The cycling conditions were as follows: 95 °C for 3 min, followed by 45 cycles of 95 °C for 10 s and 60 °C for 30 s. The primers used in this study are listed in Table S5.

### 5.13 Western Blot Analysis

Total protein was isolated from the cells and kidney tissues using RIPA lysis buffer (P0013B, Beyotime, Shanghai, China). Then, 25–30 µg of proteins per well were loaded and separated using electrophoresis on a 10%‐15% SDS‐PAGE gel at 80 V for 30 min followed by 120 V for 1 h and transferred to a nitrocellulose membrane (Bio‐Rad). After blocking with 5% skim milk in tris‐buffered saline with 1% Tween 20 (TBST), the membrane was incubated with antibodies against ANXA13, KIM‐1, p21, TβRI, p‐TβRI, Smad3, p‐Smad3, HA, Flag, and β‐actin overnight at 4 °C. The membrane was incubated with the corresponding IRDyeTM800‐conjugated secondary antibodies (Rockland Immunochemicals) at room temperature for 1 h after washing with TBST. Finally, the Odyssey Infrared Imaging System (San Diego, CA, USA) was used to detect the protein signals. The details of the antibodies used are provided in Table S6.

### 5.14 Enzyme‐Linked Immunosorbent Assay (ELISA)

Serum and urine ANXA13 levels in the participants were detected using an ELISA kit for humans (RX106972H, RUIXIN, Fujian, China) or mice (EK20380, SAB, MD, USA) according to the manufacturer's instructions.

### 5.15 Immunohistochemistry

Immunohistochemistry was performed on 3 µm paraffin‐embedded kidney sections using a microwave‐based antigen retrieval technique. The antibodies used in this study were against ANXA13, p‐Smad2/3, and KIM‐1. After incubation with the primary antibodies overnight at 4 °C, the sections were washed with PBS followed by incubation with EnVision+ System‐HRP Labeled Polymer anti‐Rabbit (4003, DAKOK) at room temperature for 1 h. Then freshly prepared DAB was added for color development. The sections were viewed using the Leica CRT6000 Light Microscope with a 0.0625 mm^2^ graticule fitted to the microscope eyepiece. The cell count was reported as cells per square millimeter (mm^2^) and analyzed using software Image‐ProPlus6.0. Positive cell or protein expression was quantified in at least 10 consecutive high‐power fields and expressed as the number of positive cells per square millimeter or as the percentage of positive area, as previously described [20, 21, 22].

### 5.16 Immunofluorescence

Paraffin‐embedded kidney sections treated with the microwave‐based antigen retrieval technique were incubated with primary antibodies against ANXA13 (25153‐1‐AP, Proteintech, Wuhan, China) or GFP (50430‐2‐AP, Proteintech, Wuhan, China) overnight at 4 °C. On the second day, the sections were washed and incubated with EnVision+ System‐HRP Labeled Polymer anti‐rabbit (4003, DAKO) at room temperature for 1 h. Fluorescence signals were developed using Alexa Fluor 555 or 488 tyramide (Invitrogen, Carlsbad, CA, USA). For tubule identification, the sections were stained with tubule‐specific markers (Vector Labs, San Francisco, CA, USA) for 10 min. Nuclei were counterstained with DAPI‐containing anti‐fade mounting medium (P0131; Beyotime, Shanghai, China). Images were captured using a Leica CRT6000 fluorescence microscope and analyzed using the Image‐Pro Plus 6.0 software.

### 5.17 Single‐Cell RNA Library Construction and Sequencing

Kidney samples obtained from three sham mice and three IRI‐AKI mice were used for single‐cell suspension preparation. The scRNA‐seq libraries of the kidney were prepared using the DNBelab C4 system as previously described by Liu et al. [46]. Briefly, single‐cell suspensions (10 000 cells/sample) were subjected to a series of steps, including droplet generation, emulsion breakage, mRNA capture, reverse transcription, cDNA amplification, and purification, resulting in barcoded scRNA‐seq libraries. Short cDNA fragments of 250–400 bp were produced by shearing, and indexed sequencing libraries were prepared according to the manufacturer's instructions. Libraries were assessed using a Qubit dsDNA Assay Kit (Thermo Fisher Scientific). All libraries were sequenced using paired‐end sequencing on the MGI DNBSEQ‐T1 sequencing platform at the China National GenBank (Shenzhen, China).

### 5.18 scRNA‐Seq Data Processing and Annotation of Cell Types

The sequencing data were filtered and demultiplexed using PISA (v0.4; https://github.com/shiquan/PISA). The resulting reads were then aligned to the mouse genome reference (mm10) using STAR (version 2.5.3) [51]. Subsequently, PISA was utilized to calculate the gene unique molecular identifiers (UMI) count of the cells and generate a gene × cell UMI count matrix. The resulting gene expression matrix was then used for cell clustering analysis with Seurat (version 4.1.0) [52]. Cells with fewer than 500 genes (UMI >0) and >20% UMI originating from the mitochondrial genome were identified as low‐quality cells. The DoubletFinder R package [53] was used to remove the doublets. A total of 23 303 cells were obtained after quality control (4230 UMI and 2165 genes were detected per cell on average). The gene count matrix was normalized using log1p. Next, the top 2000 highly variable genes were selected for principal component analysis. The 25 dimensions of harmony reduction were used to perform Louvain clustering and uniform manifold approximation and projection‐based visualization. Cell types were determined based on previously reported markers [54, 55].

### 5.19 RNA‐Seq

For RNA‐seq, three independent samples from each group (sham, IRI‐EV, and IRI‐ANXA13) were analyzed. Total RNA was extracted from the kidney tissues of The three groups using TRIzol Reagent (Magen) according to the manufacturer's instructions. Qualified samples were used for the library construction. Paired‐end libraries were prepared using an ABclonal mRNA‐seq Lib Prep Kit (ABclonal, China) following the manufacturer's instructions. Library quality was assessed using an Agilent Bioanalyzer 4150 system and sequenced on an Illumina NovaSeq 6000, generating 150 bp paired‐end reads. Data generated from the Illumina platform were subjected to bioinformatics analysis using an in‐house pipeline from Shanghai Applied Protein Technology.

DESeq2 (http://bioconductor. org/packages/release/bioc/html/DESeq2. html) was used for differential expression analysis, and DEGs with | log2FC | > 1 and Padj <0.05 were considered significantly differentially expressed. The DEGs between the IRI‐EV and IRI‐ANXA13 groups were identified and used for GO enrichment analyses. The ClusterProfiler R software package was used for GO functional enrichment analysis of DEGs. GO function was considered significantly enriched when *p*<0.05.

### 5.20 Cell Cycle Analysis by Flow Cytometry

To assess the effect of ANXA13 on the cell cycle of HK‐2 cells, a cell cycle analysis kit (4abio, FXP0211) was used, according to the manufacturer's instructions. Briefly, HK‐2 cells were fixed with 70% ethanol at 4 °C overnight. Subsequently, the cells were washed three times with cold PBS and incubated with propidium iodide containing 1% RNaseA at 37 °C for 30 min in the dark. The cells were washed twice, resuspended in PBS, and analyzed using a FACSCalibur flow cytometer (Beckman Coulter).

### 5.21 Molecular Docking

Protein structures were obtained from the UniProt database, and water molecules were removed using PyMOL 2.3.0. Protein–protein docking was performed using ZDOCK 3.0.2, and the lowest binding energy result was selected as the best docking model. The docking results were visualized and displayed as 3D diagrams using PyMOL 2.4.

### 5.22 Statistical Analysis

All data are reported as mean ± SD and were analyzed using GraphPad Prism 8.0 (GraphPad Software, San Diego, CA, USA). A two‐side paired t‐test was used for the paired samples (AKI vs. recovery phase), and a two‐side unpaired t‐test was used for two independent samples. The correlations between serum ANXA13 or urinary ANXA13 levels and SCr levels were assessed using simple linear regression analysis. When comparing one factor among multiple groups, the normality of the data distribution was assessed using the Shapiro‐Wilk test. For variables with a normal distribution (*p*>0.05), homogeneity of variances across groups was verified using Bartlett's test. One‐way analysis of variance (ANOVA) with two‐side Tukey's post hoc test was used for groups with equal variance (*p*>0.05), whereas two‐side Dunnett's post hoc test was used for groups with unequal variance (*p*<0.05). Two‐way ANOVA with two‐side Tukey's post hoc test was performed to compare different parameters between the two genotypes. Statistical significance was set at *p* <0.05.

## Author Contributions

J.L., C.W., and Y.Z. contributed equally to this study. J.L. performed the experiments, analyzed the data, and wrote the manuscript; C.W. and Y.Z. performed the experiments and collected and analyzed clinical data. Z.L. and W.Y. collected and analyzed the clinical data. Y.Z. collected and analyzed scRNA‐seq data. G.X. performed the experiments. X.‐R.H. and L.Z. supervised the experiments, and A.L. polished the manuscript. J.C. and T.Y. designed and supervised the clinical study and revised the manuscript. H.‐Y. L. designed and supervised the experiments, and revised the manuscript.

## Funding

National Natural Science Foundation of China 82070709, 82100723, 82470711, President Foundation of the Third Affiliated Hospital of Southern Medical University YQ202205, Natural Science Foundation of Guangdong Province of China 2025A1515012766.

## Conflicts of Interest

All authors declare no conflicts of interest.

## Ethics Statement

All animal experimental protocols were approved by the Animal Ethics Experimentation Committee of the Chinese University of Hong Kong (20‐243‐MIS).

## Consent

Written informed consent was obtained from each participant, and the clinical study was approved by the Ethics Committee of Southern Medical University (2023‐059).

## Supporting information




**Supporting File**: advs73464‐sup‐0001‐SuppMat.docx.

## Data Availability

All data associated with this study are provided in the article and the Supporting Information. RNA‐seq data were deposited in the Gene Expression Omnibus database (accession number GSE269541). Single‐cell RNA‐seq data from mice were deposited at the OMIX, China National Center for Bioinformation/Beijing Institute of Genomics, Chinese Academy of Sciences (https://ngdc.cncb.ac.cn/omix; accession number OMIX006672). Additional scRNA‐seq data of humans were derived from the following resources available in the public domain: The Human Protein Atlas.
