# Supplementary material for: Annexin A13 Protects Against Acute Kidney Injury by Inactivating TGF‐β/Smad3 Signaling
Source: Adv Sci (Weinh). 2026 Jan 4;13(10):e04356. doi: 10.1002/advs.202504356 (PMC12915081; doi:10.1002/advs.202504356)
Supplement: Supplementary file 1 — Supporting File: advs73464‐sup‐0001‐SuppMat.docx. [file ADVS-13-e04356-s001.docx]

**Supporting Information**

**Figure S1**


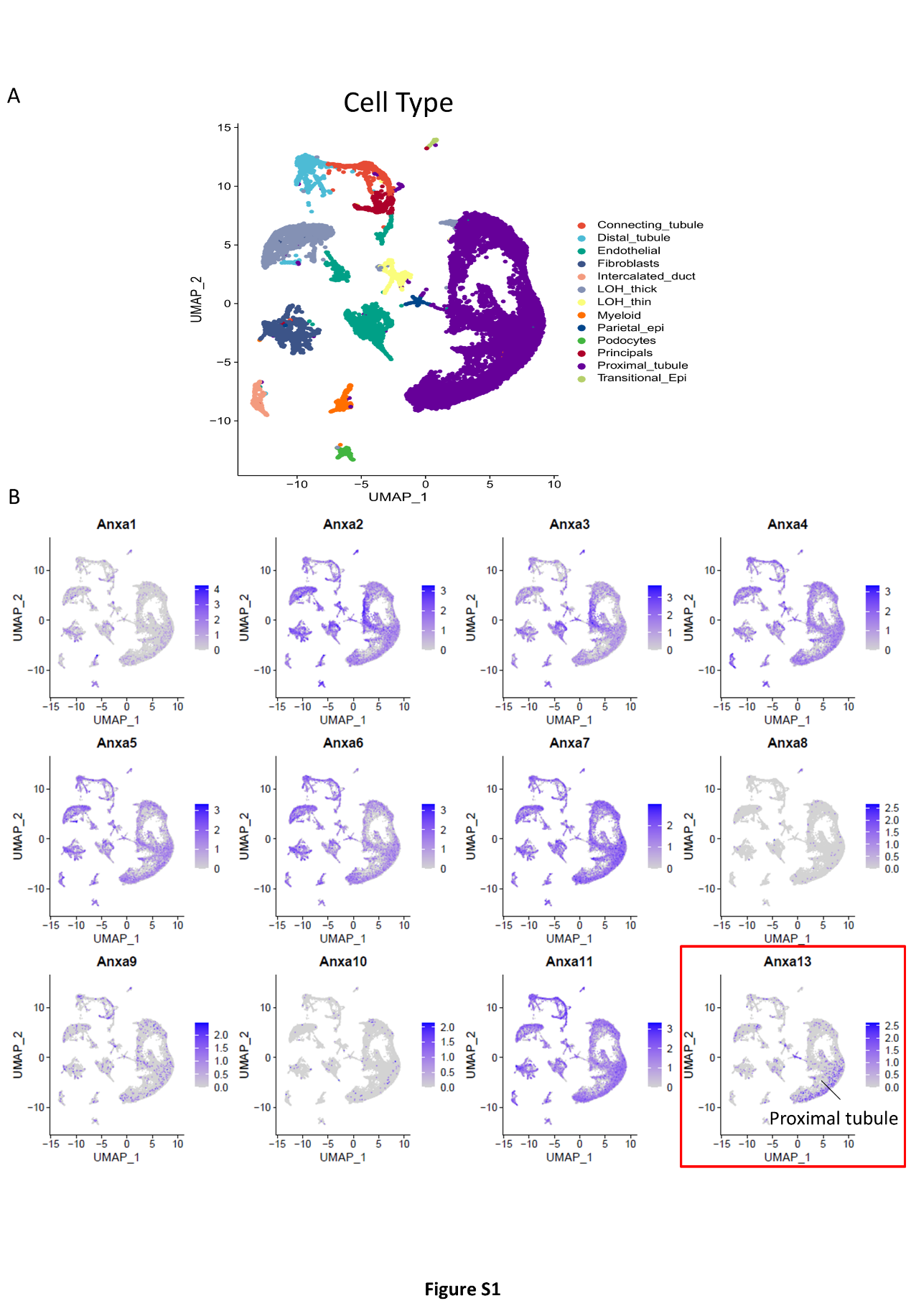


**Figure S1. Distribution and relative expression of annexin family members in mouse kidneys.** (A) Annotation of kidney cell subsets. (B) Distribution and relative expression of annexins in different kidney cell subsets, which shows that ANXA13 is predominantly expressed by proximal tubular cells in the mouse kidney.

**Figure S2**


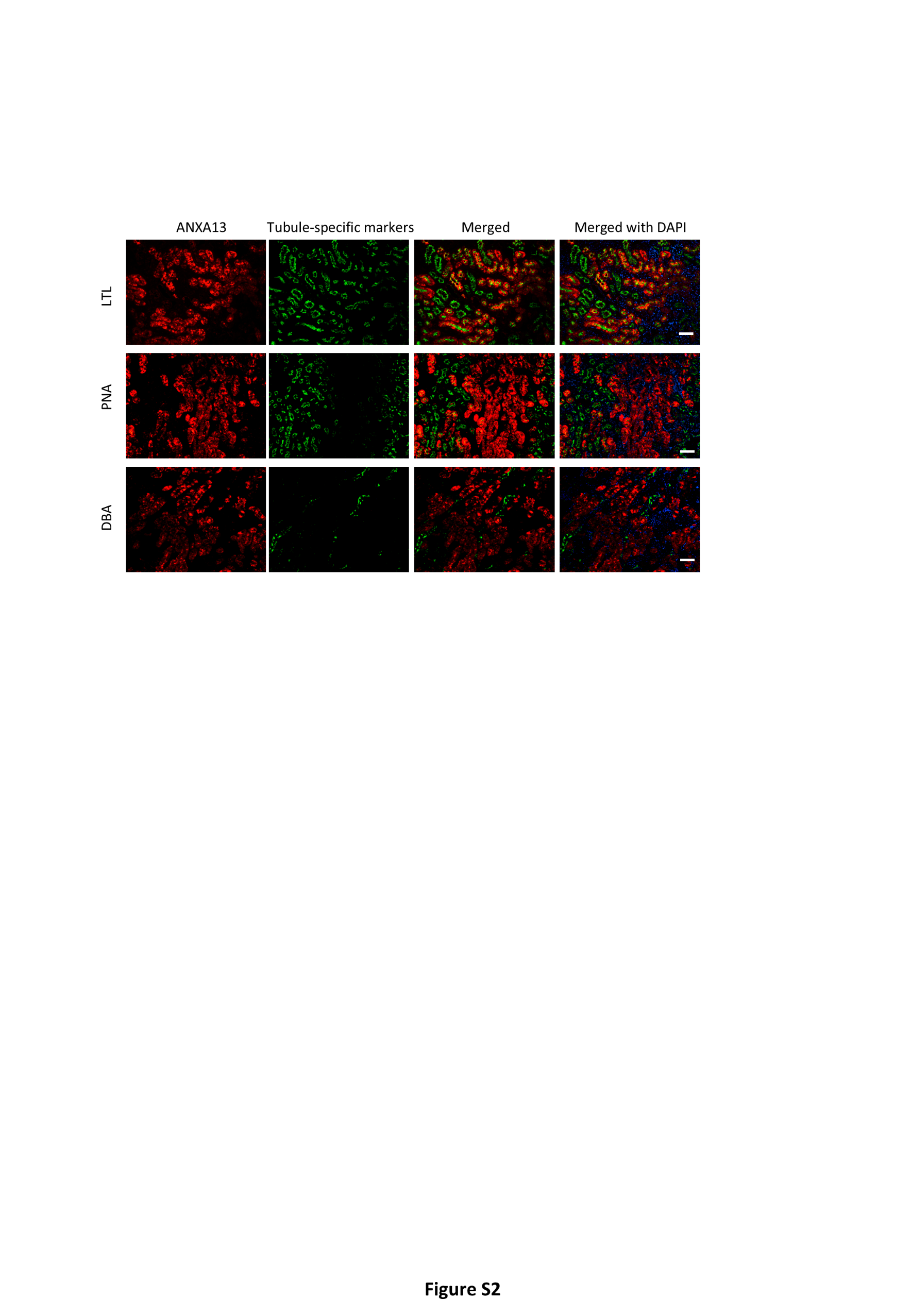


**Figure S2. Co-localization of ANXA13 and tubular-specific markers using two-color immunofluorescence.** LTL: a marker for proximal renal tubules; PNA: a marker for distal renal tubules; DBA: a marker for collecting ducts

**Figure S3**


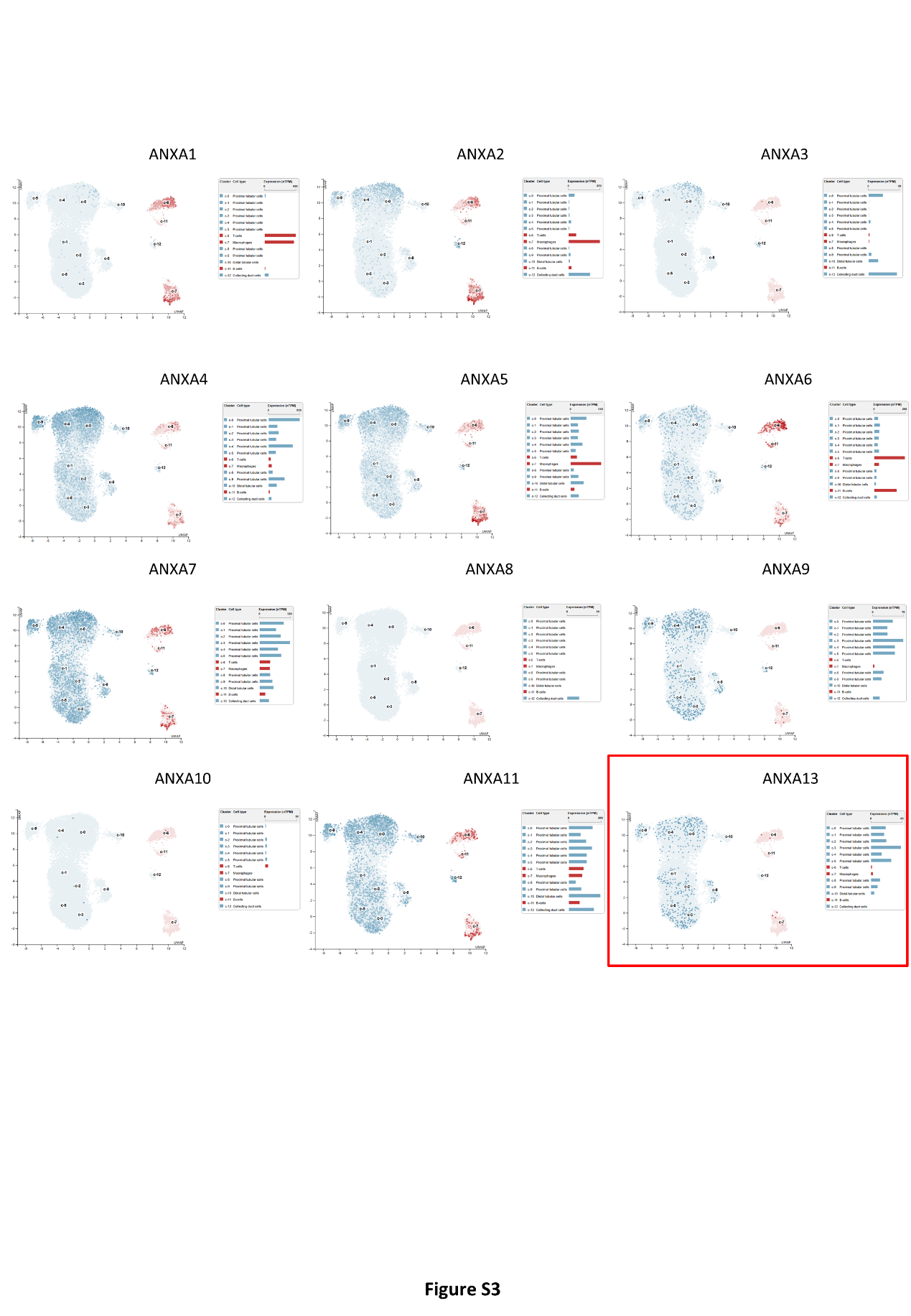


**Figure S3. Distribution and relative expression of Annexins family in human kidney (From** [**The Human Protein Atlas**](https://www.proteinatlas.org/)**, HPA)**

**Figure S4**


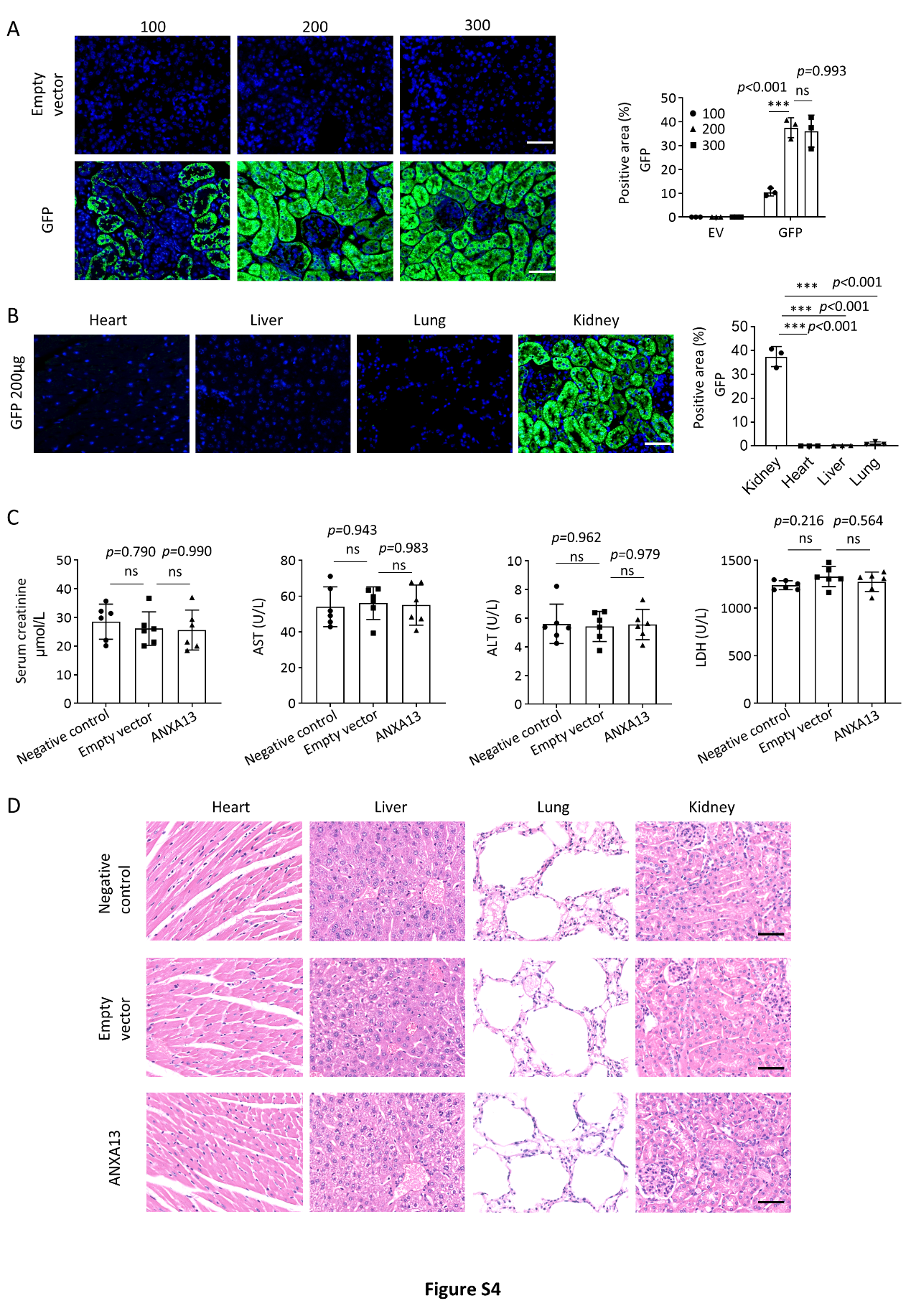


**Figure S4. Evidence for ultrasound–microbubble–mediated gene transfer efficiency, specificity, and safty. The GFP-expressing plasmids or empty vector controls at varying doses (100, 200, and 300 μg per mouse) transferred into normal mouse kidneys using the ultrasound-microbubble method. Transfection efficiency and specificity were determined by the number of GFP-expressing cells in the kidney and other organs (liver, heart, and lung) with or without ultrasound treatment. The biosafety of ultrasound-microbubble-mediated gene transfer was examined using serology and histology.** (A) Immunofluorescence and quantitative analysis of GFP-expressing cells in the kidney treated with ultrasound-microbubble-mediated GFP-expressing plasmids or empty vectors. (B) Immunofluorescence and quantitative analysis of GFP-expressing cells in the ultrasound-treated kidney versus other major organs (liver, heart, and lung) without direct ultrasound exposure. (C) Serum creatinine, AST, ALT and LDH levels in normal mice (negative control) and those treated with ultrasound-microbubble-mediated ANXA13 or empty vectors. (D)H&E staining in the ultrasound-treated kidney versus other major organs (liver, heart, and lung) without direct ultrasound exposure. Data are reported as the mean ± SD from groups of 6 mice. The statistical test is one-way ANOVA with Tukey’s test for panels A-D. Scale bars = 50 μm. Statistical analysis was performed using two-way ANOVA for B, and one-way ANOVA with Tukey’s test for C. ***p* < 0.01, ****p*< 0.001 versus GFP 100 or kidney.

**Figure S5**


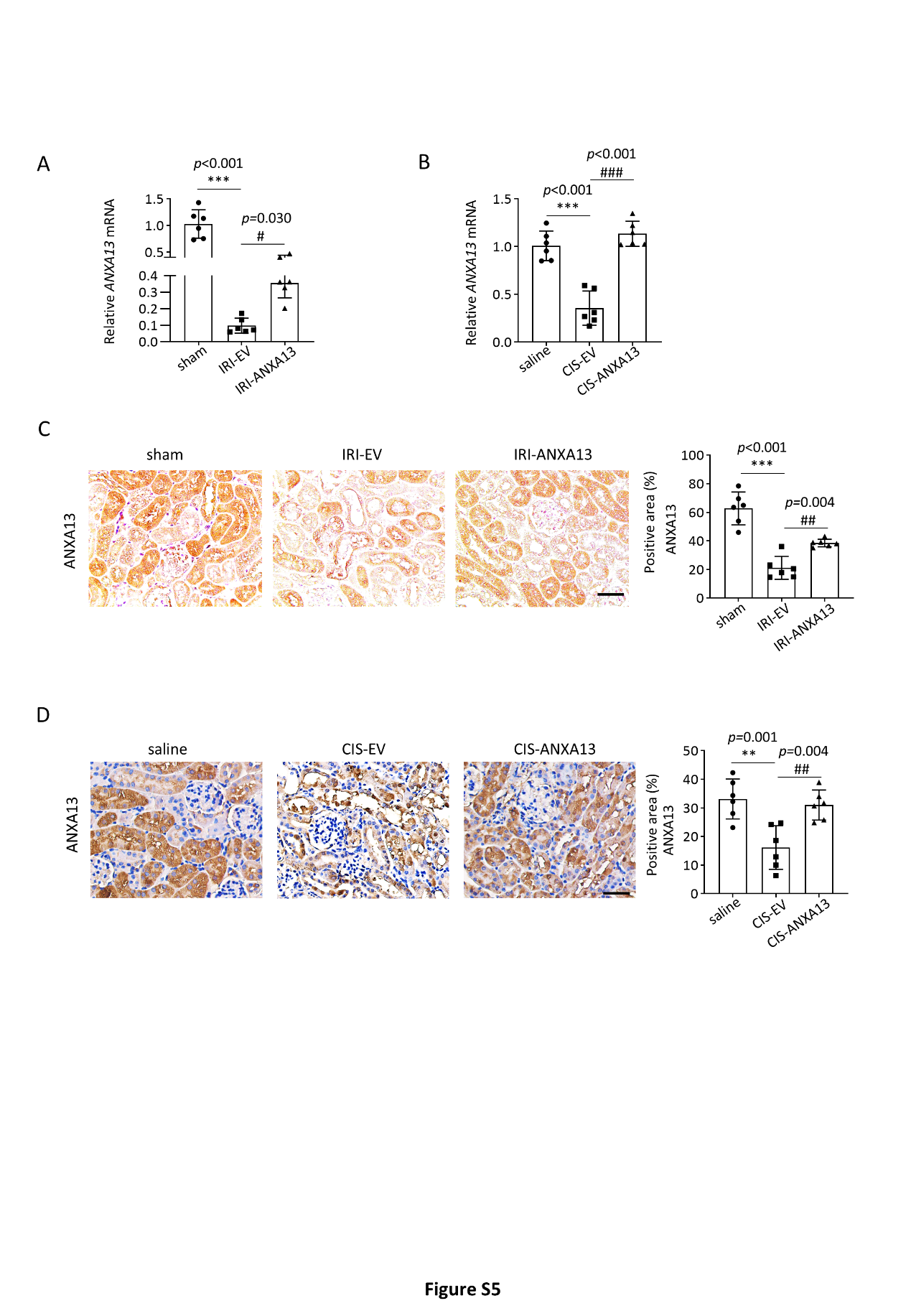


**Figure S5. Overexpression of ANXA13 in IRI- and cisplatin-induced AKI in mice.** (A, B) Relative mRNA levels of ANXA13 in IRI- and cisplatin-induced AKI mice. (C, D) Immunohistochemical staining of ANXA13 in IRI- and cisplatin-induced AKI mice. Data are reported as the mean ± SD from groups of 6 mice. Statistical analysis was performed using one-way ANOVA with Dunnett’s test for A and C and one-way ANOVA with Tukey’s test for B and D. ***p* < 0.01, ****p* < 0.001 versus sham or saline; #*p* <0.05, ##*p* < 0.01, ###*p* < 0.001 versus IRI-EV or CIS-EV; scale bars = 50 μm.

**Figure S6**


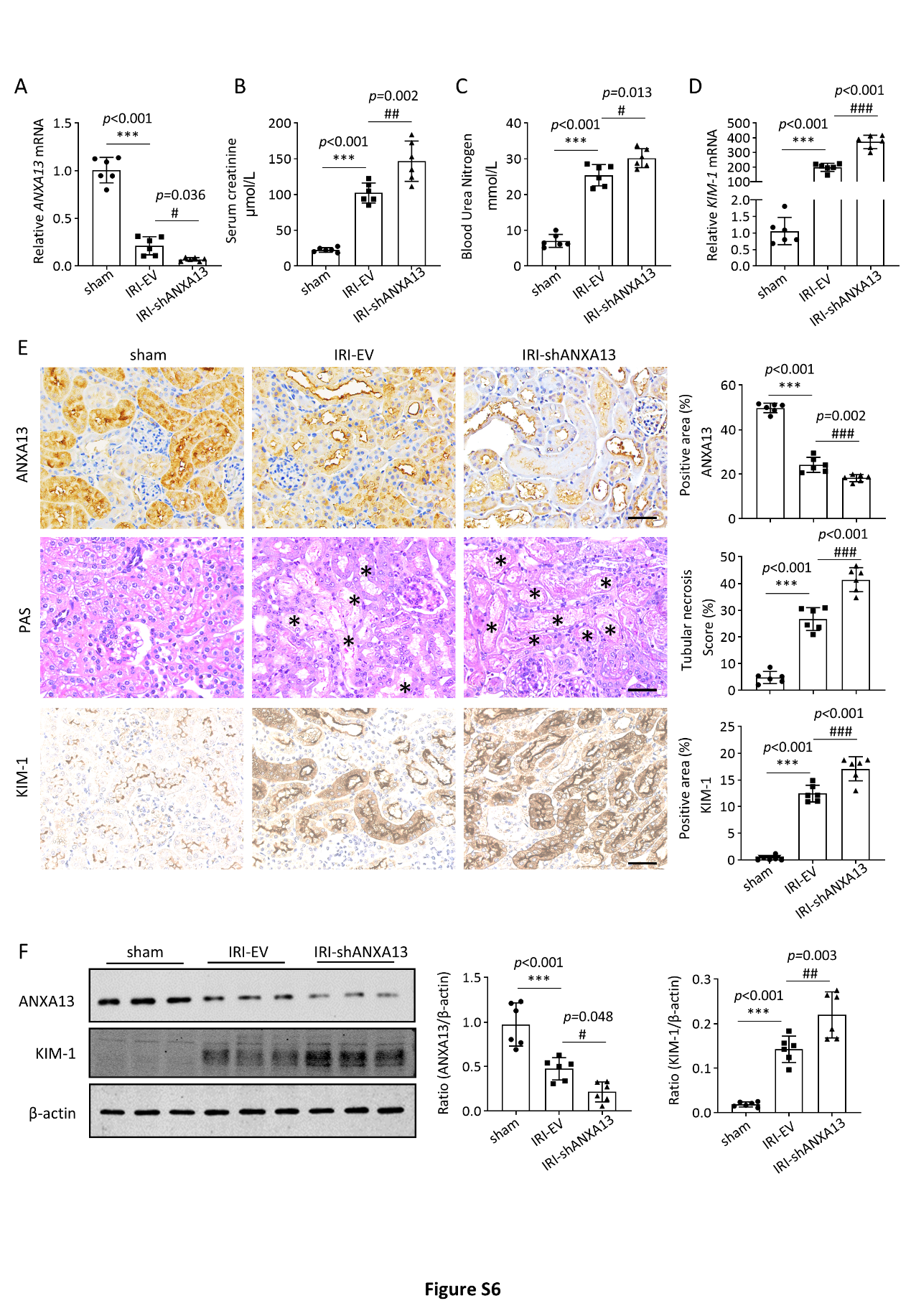


**Figure S6. Knockdown of ANXA13 promoted IRI-induced AKI in mice.** (A) Relative ANXA13 mRNA levels in IRI-induced AKI mice. (B, C) Serum creatinine and blood urea nitrogen levels in IRI-induced AKI mice. (D) Relative KIM-1 mRNA levels in IRI-induced AKI mice. (E) Immunohistochemical staining of ANXA13 and KIM-1, and PAS staining for the detection of tubular necrosis in IRI-induced AKI mice. (F) Western blot analysis of ANXA13 and KIM-1. Data are reported as the mean ± SD from groups of 6 mice. Statistical analysis was performed using one-way ANOVA with Dunnett’s test for A, B, D, E (KIM-1), F (KIM-1), and one-way ANOVA with Tukey’s test for C, E (ANXA13 and ANXA13), and F (ANXA13). ****p* < 0.001 versus sham, #*p* < 0.05, ##*p* < 0.01, ###*p* < 0.001 versus IRI-EV, scale bars = 50 μm.

**Figure S7**


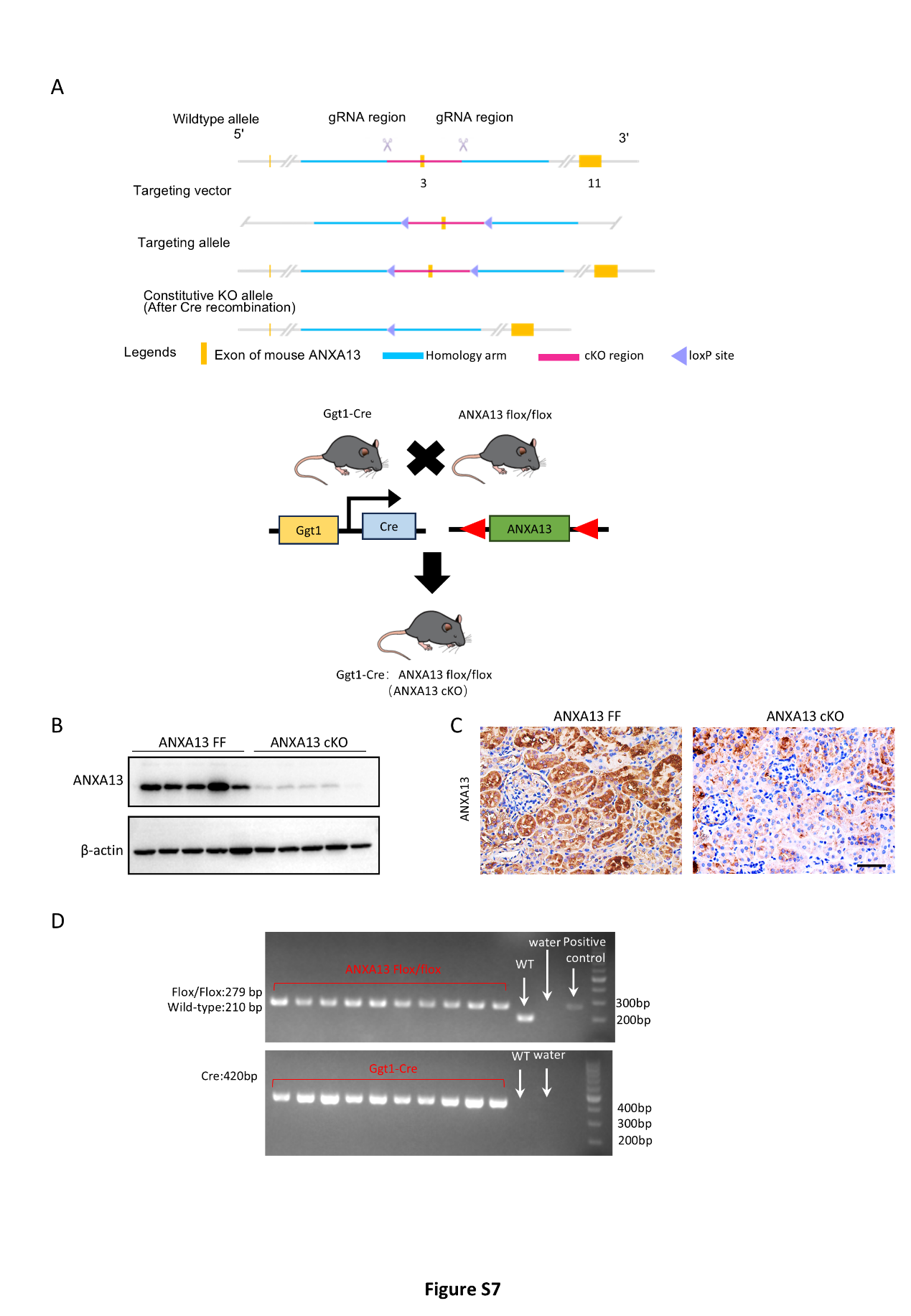


**Figure S7. Generation and Identification of *Anxa1*3 cKO mice.** (A) Schematic diagram of the generation of *Anxa1*3 Flox/Flox (FF) and *Anxa1*3 Flox/Flox/Ggt1-Cre (cKO) mice. (B) Western blot analysis of renal ANXA13 in *Anxa1*3 FF and cKO mice. (C) Immunohistochemical staining of ANXA13 in *Anxa1*3 FF and cKO mice. (D) Genotyping of *Anxa1*3 FF and cKO mice.

**Figure S8**


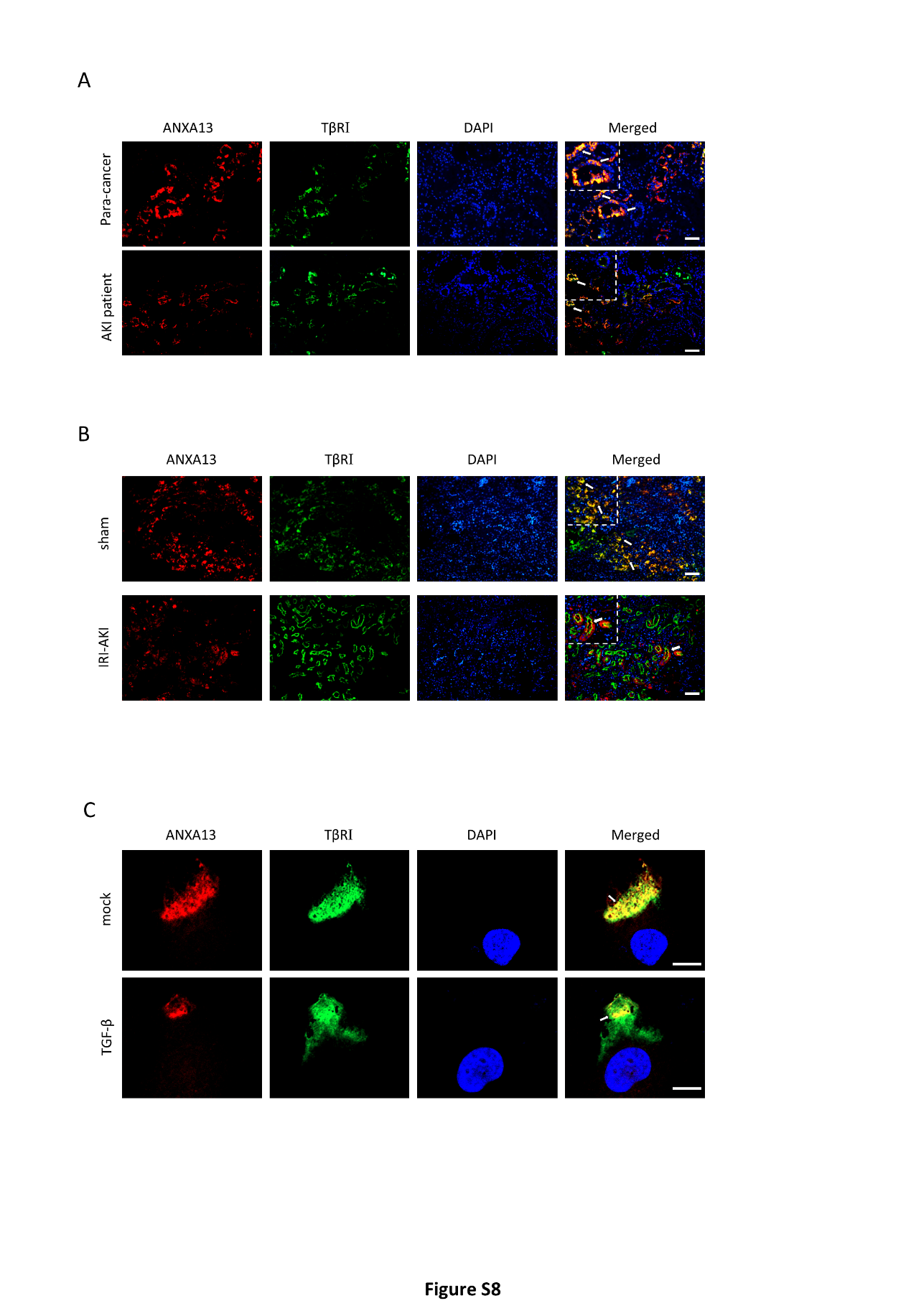


**Figure S8.** **Co-localization of ANXA13 and TβR1.** Two-color immunofluorescence shows that ANXA13 (red) is co-localized with TβRⅠ(green) in the kidneys of patients (A), mice (B), and HK-2 cells (C). White arrows show the co-localization between ANXA13 (red) and TβRⅠ(green). Scale bars = 50 μm.

**Figure S9**


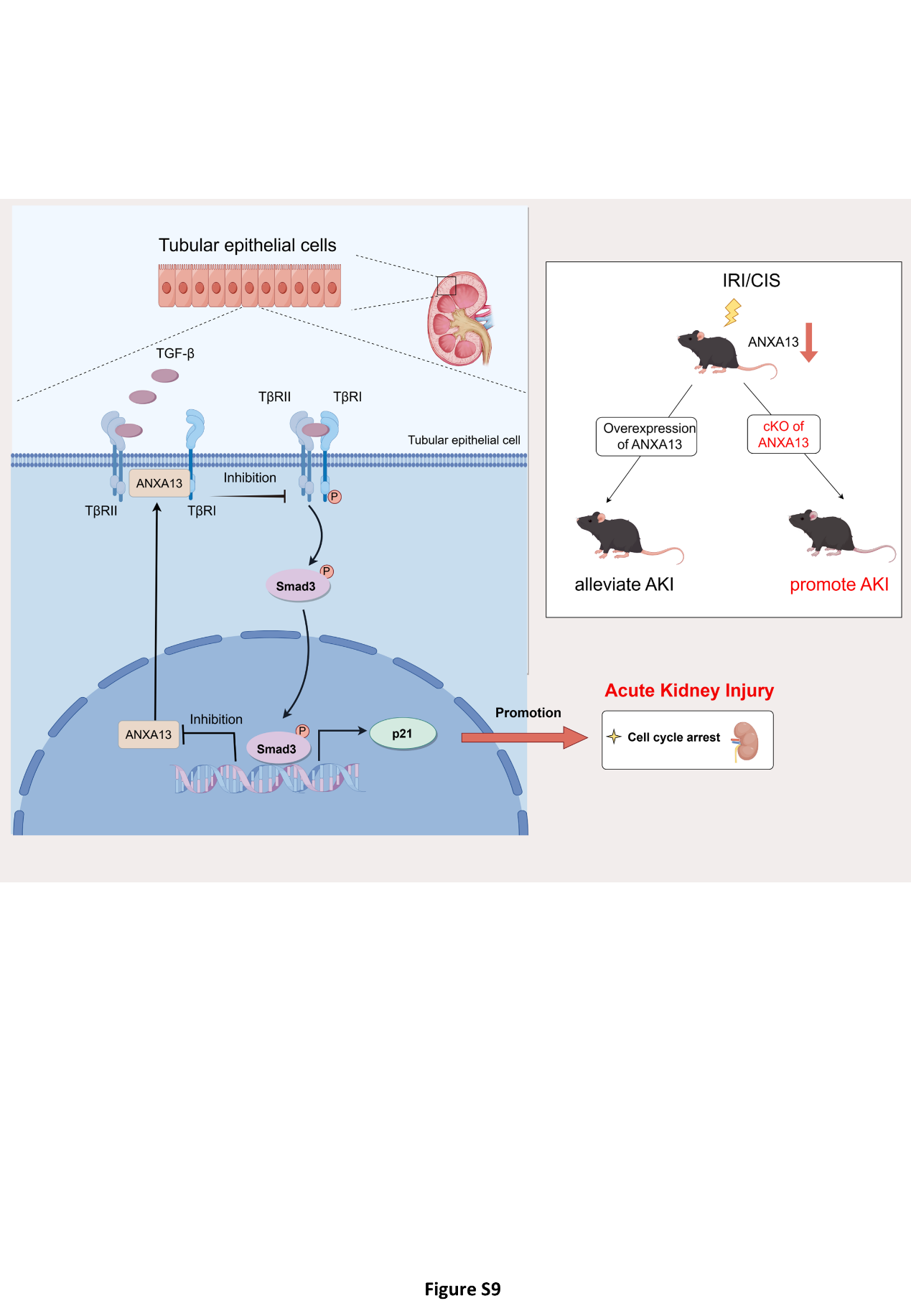


**Figure S9.** **Mechanism by which ANXA13 protects against AKI (Figdraw).** ANXA13 is negatively regulated by Smad3 and exerts its protective role in AKI by inactivating TGF-β/Smad3 signaling and Smad3-p21 cell cycle arrest pathway by binding to TβRⅠ and inhibiting the interaction between TβRⅠ and TβRⅡ.

**Table S1.** **Clinical characteristics and serum levels of ANXA13 and creatinine in patients with AKI**

| NO. | Pathologic diagnosis | Sex | Age | ANXA13-AKI | SCR-AKI |
| --- | --- | --- | --- | --- | --- |
| 1 | AKI | male | 17 | 5.88 | 160 |
| 2 | AKI | female | 78 | 1.41 | 275 |
| 3 | AKI | male | 91 | 5.48 | 151 |
| 4 | AKI | female | 87 | 2.19 | 300 |
| 5 | AKI | female | 31 | 5.73 | 125 |
| 6 | AKI | male | 67 | 6.10 | 221 |
| 7 | AKI | female | 31 | 5.47 | 165 |
| 8 | AKI | male | 30 | 2.36 | 292 |
| 9 | AKI | male | 73 | 1.47 | 266 |
| 10 | AKI | male | 67 | 5.48 | 195 |
| 11 | AKI | male | 67 | 4.55 | 229 |
| 12 | AKI | male | 18 | 3.48 | 141 |
| 13 | AKI | male | 64 | 2.50 | 198 |
| 14 | AKI | male | 84 | 3.09 | 352 |
| 15 | AKI | male | 78 | 2.66 | 168 |
| 16 | AKI | male | 19 | 3.48 | 157 |
| 17 | AKI | male | 35 | 2.08 | 425 |
| 18 | AKI | male | 38 | 3.19 | 103 |
| 19 | AKI | male | 24 | 3.31 | 376 |
| 20 | AKI | male | 66 | 4.41 | 219 |
| 21 | AKI | male | 48 | 2.99 | 397 |
| 22 | AKI | female | 35 | 3.16 | 130 |
| 23 | AKI | male | 78 | 2.46 | 165 |
| 24 | AKI | male | 35 | 4.93 | 121 |
| 25 | AKI | female | 86 | 2.87 | 472 |
| 26 | AKI | male | 58 | 3.75 | 179 |
| 27 | AKI | male | 32 | 4.51 | 142 |
| 28 | AKI | female | 65 | 3.27 | 201 |
| 29 | AKI | female | 82 | 4.57 | 247 |
| 30 | AKI | female | 53 | 2.81 | 111 |
| 31 | AKI | male | 27 | 2.66 | 266 |
| 32 | AKI | male | 51 | 3.10 | 304 |
| 33 | AKI | male | 26 | 4.73 | 268 |
| 34 | AKI | female | 31 | 2.67 | 314 |
| 35 | AKI | female | 56 | 2.89 | 278 |
| 36 | AKI | female | 72 | 3.21 | 432 |
| 37 | AKI | male | 56 | 3.22 | 275 |
| 38 | AKI | male | 52 | 6.92 | 101 |
| 39 | AKI | male | 66 | 3.05 | 168 |
| 40 | AKI | female | 86 | 2.87 | 472 |
| 41 | AKI | male | 72 | 2.68 | 280 |
| 42 | AKI | male | 52 | 2.44 | 417 |
| 43 | AKI | female | 57 | 2.89 | 139 |
| 44 | AKI | female | 26 | 3.35 | 150 |

Note: AKI: acute kidney injury; SCR: serum creatinine

**Table S2.** **Changes in serum levels of ANXA13 and creatinine in patients undergoing the recovery phase of AKI**

| NO. | Pathologic diagnosis | Sex | Age | Baseline-SCR | ANXA13-AKI | SCR-AKI | ANXA13-Recovery phase | SCR-Recovery phase |
| --- | --- | --- | --- | --- | --- | --- | --- | --- |
| 1 | AKI | male | 32 | 95 | 4.51 | 142 | 3.74 | 89 |
| 2 | AKI | female | 65 | 67 | 3.27 | 201 | 2.60 | 79 |
| 3 | AKI | female | 53 | 74 | 2.81 | 111 | 4.50 | 84 |
| 4 | AKI | male | 27 | 84 | 2.66 | 266 | 4.53 | 106 |
| 5 | AKI | male | 26 | 74 | 4.73 | 268 | 4.71 | 105 |
| 6 | AKI | female | 31 | 82 | 2.67 | 314 | 4.74 | 195 |
| 7 | AKI | female | 56 | 70 | 2.89 | 278 | 4.45 | 144 |
| 8 | AKI | female | 72 | 92 | 3.21 | 432 | 6.31 | 263 |
| 9 | AKI | male | 56 | 96 | 3.22 | 275 | 3.91 | 93 |
| 10 | AKI | male | 52 | 67 | 6.92 | 101 | 7.21 | 87 |
| 11 | AKI | male | 66 | 89 | 3.05 | 168 | 4.10 | 116 |
| 12 | AKI | female | 86 | 65 | 2.87 | 472 | 3.29 | 172 |
| 13 | AKI | male | 72 | 86 | 2.68 | 280 | 4.02 | 95 |
| 14 | AKI | male | 52 | 97 | 2.44 | 417 | 3.90 | 281 |
| 15 | AKI | female | 57 | 91 | 2.89 | 139 | 3.37 | 101 |

Note: AKI: acute kidney injury; SCR: serum creatinine

**Table S3.** **Urinary levels of ANXA13 in patients with AKI**

| No. | sex | age | A13 | UCR | ANXA13/UCR-AKI | SCR-AKI |
| --- | --- | --- | --- | --- | --- | --- |
| 1 | male | 60 | 1.15 | 6.00 | 0.19 | 265 |
| 2 | female | 63 | 1.26 | 2.90 | 0.44 | 580 |
| 3 | male | 64 | 1.79 | 7.50 | 0.24 | 198 |
| 4 | female | 67 | 1.08 | 8.50 | 0.13 | 114 |
| 5 | male | 58 | 1.58 | 6.90 | 0.23 | 179 |
| 6 | male | 42 | 1.26 | 5.20 | 0.24 | 164 |
| 7 | female | 82 | 1.36 | 4.70 | 0.29 | 250 |
| 8 | male | 67 | 1.34 | 3.60 | 0.37 | 195 |
| 9 | male | 68 | 1.48 | 4.53 | 0.33 | 438 |
| 10 | male | 73 | 1.68 | 5.90 | 0.28 | 266 |
| 11 | male | 30 | 1.04 | 7.40 | 0.14 | 218 |
| 12 | male | 67 | 1.20 | 10.50 | 0.11 | 221 |
| 13 | female | 87 | 1.25 | 6.08 | 0.21 | 300 |
| 14 | female | 26 | 1.62 | 8.70 | 0.19 | 92 |
| 15 | female | 65 | 1.18 | 3.80 | 0.31 | 101 |
| 16 | female | 78 | 1.24 | 8.98 | 0.14 | 153 |
| 17 | male | 17 | 1.41 | 4.10 | 0.34 | 160 |
| 18 | male | 67 | 1.32 | 7.67 | 0.17 | 229 |
| 19 | female | 65 | 1.51 | 8.99 | 0.17 | 201 |
| 20 | male | 51 | 1.73 | 4.21 | 0.41 | 367 |
| 21 | male | 26 | 1.05 | 7.75 | 0.13 | 274 |
| 22 | male | 66 | 1.52 | 6.06 | 0.25 | 89 |
| 23 | male | 84 | 1.66 | 4.15 | 0.40 | 352 |
| 24 | male | 78 | 2.16 | 5.15 | 0.42 | 168 |
| 25 | male | 78 | 1.69 | 3.43 | 0.49 | 165 |
| 26 | female | 58 | 1.92 | 3.37 | 0.57 | 811 |
| 27 | male | 52 | 1.37 | 2.21 | 0.62 | 417 |
| 28 | male | 53 | 1.58 | 5.02 | 0.32 | 147 |
| 29 | male | 27 | 1.70 | 3.58 | 0.47 | 266 |
| 30 | male | 52 | 1.13 | 2.40 | 0.47 | 101 |
| 31 | female | 62 | 1.81 | 9.70 | 0.19 | 137 |
| 32 | female | 56 | 1.23 | 4.29 | 0.29 | 218 |
| 33 | female | 72 | 0.06 | 1.72 | 0.03 | 700 |
| 34 | male | 42 | 0.51 | 5.80 | 0.09 | 85 |
| 35 | male | 72 | 1.22 | 1.66 | 0.73 | 280 |
| 36 | male | 58 | 0.08 | 5.10 | 0.02 | 254 |
| 37 | male | 22 | 0.13 | 1.02 | 0.13 | 230 |
| 38 | male | 52 | 1.37 | 2.20 | 0.62 | 538 |
| 39 | male | 35 | 0.40 | 2.51 | 0.16 | 327 |
| 40 | male | 73 | 0.06 | 0.93 | 0.06 | 139 |

Note: AKI: acute kidney injury; SCR: serum creatinine; UCR: urine creatinine

**Table S4. Changes in urinary ANXA13 levels in patients during the recovery phase of AKI**

| No. | sex | age | Baseline-SCR | A13 | UCR | A13/UCR-AKI | Scr-AKI | A13 | UCR | A13/UCR-Recovery phase | Scr-Recovery phase |
| --- | --- | --- | --- | --- | --- | --- | --- | --- | --- | --- | --- |
| 1 | male | 27 | 84 | 1.70 | 3.58 | 0.47 | 266 | 1.42 | 3.78 | 0.38 | 106 |
| 2 | male | 52 | 65 | 1.13 | 2.40 | 0.47 | 101 | 1.28 | 7.74 | 0.17 | 89 |
| 3 | female | 62 | 75 | 1.81 | 9.70 | 0.19 | 137 | 2.96 | 19.40 | 0.15 | 87 |
| 4 | female | 56 | 70 | 1.23 | 4.29 | 0.29 | 218 | 1.07 | 9.10 | 0.12 | 113 |
| 5 | female | 72 | 70 | 0.06 | 1.72 | 0.03 | 700 | 0.06 | 1.68 | 0.03 | 431 |
| 6 | male | 42 | 79 | 0.51 | 5.80 | 0.09 | 185 | 0.08 | 8.40 | 0.01 | 69 |
| 7 | male | 72 | 86 | 1.22 | 1.66 | 0.73 | 280 | 2.13 | 4.69 | 0.45 | 95 |
| 8 | male | 58 | 96 | 0.08 | 5.10 | 0.02 | 254 | 0.06 | 3.10 | 0.02 | 141 |
| 9 | male | 22 | 106 | 0.13 | 1.02 | 0.13 | 230 | 0.48 | 3.80 | 0.13 | 81 |
| 10 | male | 35 | 98 | 0.40 | 2.51 | 0.16 | 327 | 0.06 | 2.10 | 0.03 | 85 |
| 11 | male | 73 | 92 | 0.06 | 0.93 | 0.06 | 139 | 0.16 | 9.20 | 0.02 | 104 |

Note: AKI: acute kidney injury; Scr: serum creatinine; Ucr: urine creatinine

**Table S5. Primers used in this study**

| **1. Primers for qPCR** | | | |
| --- | --- | --- | --- |
| Gene | species | Forward (5’-3’) | Reverse (5’-3’) |
| ANXA13 | Human | GCTAAAGCGAGCAGTCCTCAG | GTCCTGCCCGATAAGATTTCAA |
| p21 | Human | AGTCAGTTCCTTGTGGAGCC | CGCAGAAACACCTGTGAACG |
| β-actin | Human | TGATCTTCATTGTGCTGGGTG | CCTTCCTGGGCATGGAGTC |
| ANXA13 | Mouse | ATTGTGACCAGAGCAGAGGTG | TACCAGCAGTTTCCGGAAGTC |
| KIM-1 | Mouse | ACATATCGTGGAATCACAACGAC | ACAAGCAGAAGATGGGCATTG |
| p21 | Mouse | AGGCATATCTAGGCACTTGC | CCACACACCATAGAATGCTC |
| β-actin | Mouse | CAGAGGCATACAGGGACAGC | GCCAACCGTGAAAAGATGAC |
| **2. Primers for plasmid construction** | | | |
| Gene | species | Forward (5’-3’) | Reverse (5’-3’) |
| Flag-ANXA13 | Human | cttggtaccgagctcggatccATGGGCAATCGTCATGCTAAA | ccacactggactagtggatccTCAGTGCAAGAGGGCTACTAGCA |
| Flag-ANXA13 (1-241) | Human | cttggtaccgagctcggatccATGGGCAATCGTCATGCTAAA | ccacactggactagtggatccTCATCTCACGAGAGTTAAATAGGCC |
| Flag-ANXA13 (1-165) | Human | cttggtaccgagctcggatccATGGGCAATCGTCATGCTAAA | ccacactggactagtggatccTCACACGTCATCTCCTTCATTGC |
| Flag-ANXA13 (1-91) | Human | cttggtaccgagctcggatccATGGGCAATCGTCATGCTAAA | ccacactggactagtggatccTCAGGCGTACTCGCTGGG |
| GST-ANXA13 | Human | ttccaggggcccctgggatccATGGGCAATCGTCATGCTAAA | acccgggaattccggggatccTCAGTGCAAGAGGGCTACTAGCA |
| HA-TβRⅠ | Human | catcattttggcaaagaattcATGGAGGCGGCGGTCGCT | tgcatcgatgagctcgaattcTTACATTTTGATGCCTTCCTGTTG |
| HA-TβRⅠⅠ | Human | catcattttggcaaagaattcATGGGTCGGGGGCTGCT | tgcatcgatgagctcgaattcCTATTTGGTAGTGTTTAGGGAGCCG |
| HA-Smad2 | Human | catcattttggcaaagaattcATGTCGTCCATCTTGCCATTCA | tgcatcgatgagctcgaattcTGACATGCTTGAGCAACGCA |
| HA-Smad3 | Human | catcattttggcaaagaattcATGTCGTCCATCCTGCCTTTC | tgcatcgatgagctcgaattcCTAAGACACACTGGAACAGCGG |
| HA-TβRⅠ-ECD | Human | catcattttggcaaagaattcATGGAAGCCGCTGTGGCC | ttggcagagggaaaaagatctTCAAGCGTAGTCTGGGACGTC |
| HA-TβRⅠ-ETD | Human | catcattttggcaaagaattcTGATGGAGGCGGCGGTCG | tgcatcgatgagctcgaattcGATATAGACCATCAACATGAGTGAGATG |
| HA-TβRⅠ-ICD | Human | catcattttggcaaagaattcTGCCACAACAGAACCGTGATC | ttggcagagggaaaaagatctTCAAGCGTAGTCTGGGACGTC |

Note: ECD: extracellular domain; ETD: extracellular and transmembrane domain; ICD: intracellular domain

**3. Primers for genotyping of ANXA13 cKO mice and CHIP**

|  | Forward (5’-3’) | Reverse (5’-3’) |
| --- | --- | --- |
| Flox | ATTTGGGTTTCAATCTTACCTGTTC | ACCTGTCTATGACCACACCAC |
| Ggt1-Cre | CATCACATCAGGCACCCCAGAA | GAACATCTTCAGGTTCTGCGGGA |
| ANXA13 | GCATCACACAAGTAAGGTACAGG | TCAAATTGAACACACAGAAGGAAA |

**Table S6. Antibodies used in this study**

| Name | Use | Concentration | species | Number | Company |
| --- | --- | --- | --- | --- | --- |
| ANXA13 | WB | 1:2000 | Mouse | 25153-1-AP | Proteintech |
|  | WB | 1:1000 | Human | ab151517 | abcam |
|  | IHC, IF | 1:100 | Mouse | 25153-1-AP | Proteintech |
| KIM-1 | WB | 1:1000 | Mouse | AF1817 | R&D |
|  | IHC | 1:100 | Mouse | AF1817 | R&D |
| p-Smad3 | WB | 1:1000 | Mouse， Human | ab52903 | abcam |
|  | IHC | 1:100 | Mouse | 600-401-919 | Rockland |
| Smad3 | WB | 1:1000 | Mouse， Human | PA5-32588; MA5-14939 | Invitrogen |
|  | ChIP | 1:50 | Human | C67H9 | CST |
| p21 | WB | 1:1000 | Mouse | ab188224 | abcam |
|  | WB | 1:1000 | Human | ab109520 | abcam |
| PCNA | IHC | 1:100 | Mouse | sc-7907 | Santa Cruz |
| TβRI | WB | 1:1000 | Mouse， Human | ab288303 | Abcam |
| p-TβRI | WB | 1:1000 | Mouse， Human | PA5-40298 | Invitrogen |
| Flag | WB | 1:1000 | Human | F3165 | Sigma |
| HA | WB | 1:1000 | Human | H6908 | Sigma |
| GST | WB | 1:1000 | Human | AE077 | abclonal |
| β-actin | WB | 1:1000 | Mouse, Human | sc-69879 | Santa Cruz |
